# Dentin defects caused by a *Dspp*^−1^ frameshift mutation are associated with the activation of autophagy

**DOI:** 10.1038/s41598-023-33362-1

**Published:** 2023-04-19

**Authors:** Tian Liang, Charles E. Smith, Yuanyuan Hu, Hong Zhang, Chuhua Zhang, Qian Xu, Yongbo Lu, Ling Qi, Jan C.-C. Hu, James P. Simmer

**Affiliations:** 1 Department of Biologic and Materials Sciences, University of Michigan School of Dentistry, 1011 North University, Ann Arbor, MI 48109-1078 USA; 2 Department of Anatomy & Cell Biology, Faculty of Medicine & Health Sciences, McGill University, Montreal, QC Canada; 3 Department of Biomedical Sciences and Center for Craniofacial Research and Diagnosis, Texas A&M University College of Dentistry, 3302 Gaston Ave., Dallas, TX 75246 USA; 4 Department of Molecular & Integrative Physiology, Department of Internal Medicine, Division of Metabolism, Endocrinology and Diabetes, University of Michigan Medical School, 1000 Wall St., Ann Arbor, MI 48105 USA

**Keywords:** Dental diseases, Disease model, Autophagy

## Abstract

Dentin sialophosphoprotein (*DSPP*) is primarily expressed by differentiated odontoblasts (dentin-forming cells), and transiently expressed by presecretory ameloblasts (enamel-forming cells). Disease-causing *DSPP* mutations predominantly fall into two categories: 5’ mutations affecting targeting and trafficking, and 3’ − 1 frameshift mutations converting the repetitive, hydrophilic, acidic C-terminal domain into a hydrophobic one. We characterized the dental phenotypes and investigated the pathological mechanisms of *Dspp*^P19L^ and *Dspp*^−1fs^ mice that replicate the two categories of human *DSPP* mutations. In *Dspp*^P19L^ mice, dentin is less mineralized but contains dentinal tubules. Enamel mineral density is reduced. Intracellular accumulation and ER retention of DSPP is observed in odontoblasts and ameloblasts. In *Dspp*^−1fs^ mice, a thin layer of reparative dentin lacking dentinal tubules is deposited. Odontoblasts show severe pathosis, including intracellular accumulation and ER retention of DSPP, strong ubiquitin and autophagy activity, ER-phagy, and sporadic apoptosis. Ultrastructurally, odontoblasts show extensive autophagic vacuoles, some of which contain fragmented ER. Enamel formation is comparable to wild type. These findings distinguish molecular mechanisms underlying the dental phenotypes of *Dspp*^P19L^ and *Dspp*^−1fs^ mice and support the recently revised Shields classification of dentinogenesis imperfecta caused by *DSPP* mutations in humans. The *Dspp*^−1fs^ mice may be valuable for the study of autophagy and ER-phagy.

## Introduction

Dentin sialophosphoprotein (DSPP) cleavage products are the most abundant non-collagenous proteins in the dentin organic matrix^1^. During tooth development DSPP is continuously expressed by differentiated odontoblasts (dentin-forming cells) and transiently expressed by presecretory ameloblasts (enamel-forming cells)^2^. The DSPP protein is cleaved following its secretion^3^ into the N-terminal dentin sialoprotein (DSP) and the C-terminal dentin phosphoprotein (DPP)^4^. Mutations in dentin sialophosphoprotein (*DSPP*) cause non-syndromic autosomal dominant dentinogenesis imperfecta (DGI)^5,6^. Disease-causing *DSPP* mutations fall predominately into two categories: (1) 5’ mutations altering the first three amino acid residues following the signal peptide^7^, i.e. isoleucine-proline-valine (IPV), and (2) 3’ -1 frameshift mutations that replace the downstream repetitive, hydrophilic and acidic wild type (WT) DPP sequence with a longer, hydrophobic one^8–10^. For diagnostic purposes the Shields classification^11^ was recently modified to correlate the 5’ and 3’ *DSPP* mutations with a diagnosis of DGI-III and DGI-II, respectively^6^.

Previously we generated two *Dspp*-knockin mouse models: *Dspp*^P19L^ mice^12^ homologous to the p.Pro17Leu mutation in humans^6,13–15^ representing the 5’ group of *DSPP* mutations, and *Dspp*^−1fs^ mice^16^ representing the 3’ group of human *DSPP* -1 frameshift mutations. Both mice displayed dental defects inherited in an autosomal dominant pattern and showed intracellular accumulation of mutated DSPP but varied in their dental phenotypes and cell abnormalities. Molar dentin in *Dspp*^P19L^ mice is initially thinner (at 3 weeks), equals the WT at 8 weeks, and is thicker than the WT at 24 weeks^12^. The dentin contains dentinal tubules but is less mineralized than normal. The continuous dentinal tubules indicate that the original odontoblasts continue to make dentin. In this respect the odontoblast response resembles the formation of reactionary dentin^17^. The enamel shows reduced mineral density in incisors and patches of enamel dysplasia in molars^16,18^, indicating that ameloblast were altered by their transient expression of the mutated DSPP protein.

Dentin defects in *Dspp*^−1fs^ mice are more severe than those in the *Dspp*^P19L^ mice, with limited dentin deposition and the absence of dentinal tubules. The original odontoblasts do not long survive the expression of the frameshifted DSPP and are replaced by pulp progenitor cells that initiate expression of dentin matrix protein 1 (DMP1), a marker expressed by differentiating odontoblasts^16^. These cells undergo an apparent cycling of -1 frameshifted DSPP expression, cell death, and induction of pulp progenitor cells that express the -1 frameshifted DSPP. This odontoblast response resembles the formation of reparative dentin^17^. Ameloblasts seem to recover from their transient expression of *Dspp*^−1fs^ and produce enamel comparable to that of the WT^16^. Here we further investigate the cell abnormalities underlying these two phenotypes.

Accumulation of mutant/misfolded proteins in the endoplasmic reticulum (ER) causes ER stress. ER stress can trigger a variety of coordinated protein quality control mechanisms, including the unfolded protein response (UPR), ER-associated degradation (ERAD), and autophagy^19^. The UPR involves a signaling network to (1) attenuate protein translation to alleviate the protein folding load, (2) assist ER protein folding via upregulating the expression of chaperones and ER biogenesis, and (3) eliminate misfolded proteins and protein aggregates through ERAD and autophagy^20,21^. ERAD recognizes misfolded proteins in the ER, retrotranslocates them to the cytoplasm for ubiquitination then proteasomal degradation^19,22,23^. Ubiquitinated misfolded proteins, particularly protein aggregates, can trigger autophagy^24^. Autophagy starts with a membrane cistern engulfing cytoplasmic contents to form a double-membraned vacuole autophagosome, which is later fused with a lysosome to form single-membraned autolysosome^25^. By fusing with a lysosome, autophagic vacuoles gain lysosomal enzymes that can degrade the misfolded proteins and cytoplasmic organelles^25,26^. In the case of ER, aberrant proteins can be segregated in part of the ER, recognized by ER-phagy adaptors, and captured by the autophagy-lysosome system for degradation (ER-phagy)^27,28^.

Given the multiple clinical designations of inherited conditions caused by mutations in DSPP^5,6,11^ and their correlation with the severity of the patient clinical findings and their prognosis, we further investigate the disease mechanisms underlying the two distinct groups of inherited dentin disorders resulting from mutations in different regions of the same gene in *Dspp*^P19L^ and *Dspp*^−1fs^ mice.

## Results

### Odontoblast pathosis in the ***Dspp***^−1fs^ mice

**Figure 1:**
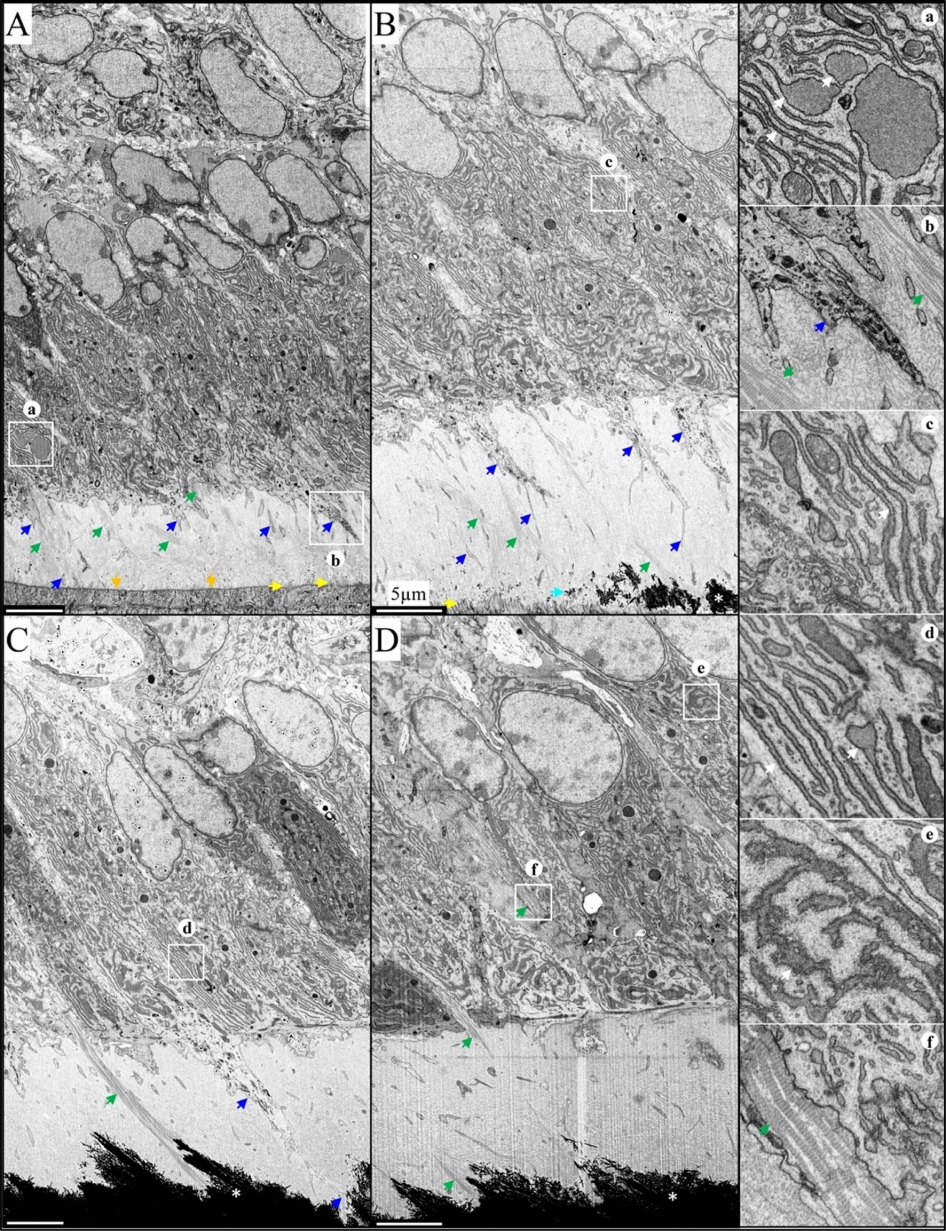
FIB-SEM of a 7-week-old Wild Type Incisor Showing Normal Odontoblast Morphology and Dentin Formation. (**A**–**D**). 5000 × FIB-SEM montages from areas -2 (A), 0 (B), S1 (C), and S2 (D) from Fig S1. (**a**–**f**). 10,000 × FIB-SEM insets of boxed areas. (**A**) The basement membrane separating ameloblasts from pre-dentin (orange arrows) is gradually degraded and penetrated by cell protrusions (yellow arrows). Note the polarized odontoblasts with odontoblastic processes (blue arrows) and collagen bundles (green arrows) between odontoblasts and in pre-dentin. (**a**) Regular and enlarged ER cisternae (white arrows) with clear membrane boundary. (**b**) Odontoblastic process (blue arrow) surrounded by matrix rich in collagen bundles (green arrows). (**B**) Dentin mineral foci (cyan arrow) in pre-dentin coalesce into a continuous layer of dentin (*). (**c**) Parallel ER cisternae (white arrow) and Golgi apparatus. (**C**) Collagen bundle spanning pre-dentin is mineralized in dentin (*). An odontoblastic process branches and extends into mineralized dentin (blue arrow). (**d**) Stacks of ER (white arrow). (**D**) Collagen bundles (green arrows) between odontoblasts. (**e**) ER (white arrow) with rough border near nucleus. (**f**) Banded collagen bundles (green arrow) between odontoblasts. Scale bars: 5 μm.

Odontoblast pathosis in homozygous *Dspp*^−1fs^ continuously growing mandibular incisors was visualized at high resolution using focused ion beam scanning electron microscopy (FIB-SEM). For comparison, FIB-SEM of WT odontoblasts and dentin formation are provided (Figs. 1, S1). Dentin formation in WT mice showed well-aligned early odontoblasts developing their characteristic columnar and polarized morphology, with the nucleus positioned proximally (Figure S1A). These odontoblasts showed abundant endoplasmic reticulum (ER), Golgi apparatus, secretory vesicles, and mitochondria (Figs S1B,C,D, 1A,B,C,D), indicative of functional secretory activity. Most of the ER cisternae were parallelly aligned in the odontoblasts, and mitochondria were consistently found nearby (Fig. 1a,c,d,e). Some ER cisternae with a clear boundary were enlarged (Fig. 1a), while some showed relatively rough borders (Fig. 1e). At the distal end, the odontoblastic processes extended from the cell bodies into the pre-dentin and dentin matrixes and branched into smaller processes, some of which almost extended to the DEJ (Fig. 1A,B,C and 1b). Collagen bundles were evident between odontoblasts and in the pre-dentin matrix (Fig. 1f). These collagen bundles extended to the distal membrane of ameloblasts (Fig. 1A,B). Mineral foci in the dentin matrix formed in close association with the collagen bundles and coalesced into the bulk of mineralized dentin (Fig. 1B,C). As the thickness of mineralized dentin expanded, a stable thickness of pre-dentin matrix was maintained by odontoblasts (Figure S1). As dentin mineralization continued, odontoblasts moved from the DEJ further into the pulp (Figure S1).

**Figure 2:**
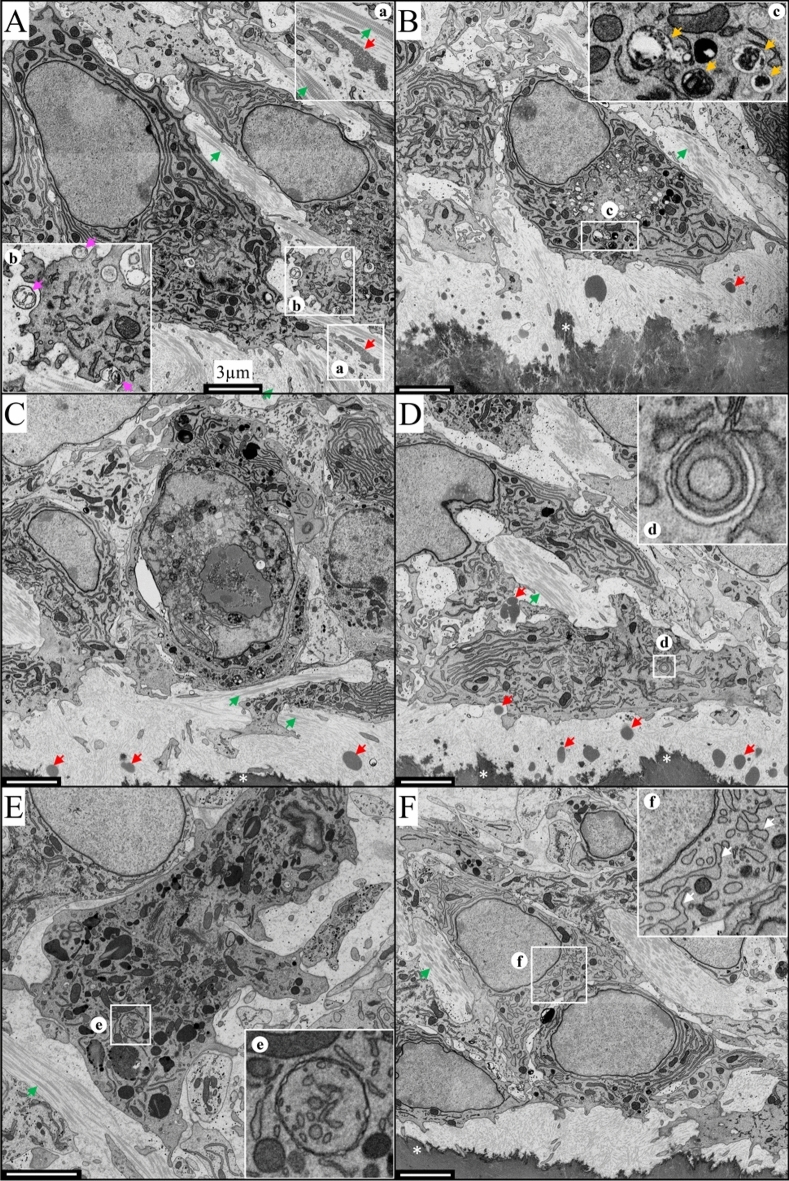
10,000 × FIB-SEM of a 7-week-old *Dspp*^−1fs/−1fs^ incisor showing odontoblast pathosis and apoptosis. (See Figure S2 for area sampling locations; see Fig. 1 and S1 for WT comparisons.) (**A**) (area -1) Odontoblasts prior to the onset of dentin mineralization were rich in endoplasmic reticulum (ER) and mitochondria and already separated by wide intercellular spaces, exposing collagen bundles (green arrows). Medium-density protein aggregates (red arrow inset (**a**)) were observed in pre-dentin and extracellular vesicles (magenta arrows inset (**b**)) containing fragmented cisternae were evident. (**B**) (area 0) Pathological odontoblasts show numerous intracellular degradative vacuoles, intercellular collagen bundles (green arrow) and extracellular medium-density plaques (red arrows). Existing pre-dentin mineralizes (*) but new pre-dentin is not deposited and thins. Vacuoles (orange arrows inset (**c**)) contained cytoplasmic material in various stages of degradation. (**C**) (area S1) Apoptotic odontoblast with intranuclear vacuoles, nuclear destruction, and severe cytoplasmic pathosis. (**D**) (area S1) Extracellular medium-density plaques (red arrows) in pre-dentin and between odontoblasts. (**d**) Autophagosome with two layers of limiting membranes and an electron-lucent cisterna. (**E**) (area S2) Degenerating odontoblast. (**e**) A double-membrane-bounded vesicle containing fragmented ER. (**F**) (area S3) Odontoblast-like cells. (**f**) Swelling ER (white arrows). Scale bars: 3 μm.

In contrast, the odontoblasts of *Dspp*^−1fs/−1fs^ mice began to lose their polarization and cellular contacts even before dentin mineral foci coalesced into a continuous mineralized layer of dentin (Figs. 2 and S2). Pathological odontoblasts lost their organization as a sheet and showed numerous intracellular vesicles that contained cytoplasmic material at various stages of degradation (Fig. 2b, c) and swollen ER (Fig. 2f). Typical autophagosomes (Fig. 2d) and double-membraned vesicles that contained fragmented ER (Fig. 2e) indicative of active autophagy, were observed in odontoblasts. Intercellular contacts between odontoblasts were loose and irregular, exposing banded collagen fibers between odontoblasts (Fig. 2), which were less evident in WT mice (Figs. 1 and S1). Odontoblastic processes were not observed in *Dspp*^−1fs/−1fs^ mice (Figure S2). Some odontoblasts showed severe cytoplasmic pathosis, intranuclear vacuoles and nuclear destruction (Fig. 2C), which are characteristic of apoptosis. Pre-dentin deposition slowed dramatically as odontoblast cellular pathosis progressively worsened, which consequently limited dentin mineralization. Dentin mineral gradually filled the existing pre-dentin space and spread up collagen bundles between odontoblasts (Figure S2). This ectopic mineral deposition between the odontoblasts was another sign of a disturbed mineralization process. Medium-density plaques were observed between odontoblasts and in the pre-dentin matrix (Figs. 2A,B,C,D, 2a, and S2), which corresponded to previously reported DSP-positive extracellular protein aggregates^16^.

To gain a better understanding of the nature of the odontoblast pathosis observed by electron microscopy, we conducted a series of immunohistochemical studies using light microscopy.

### Loss of cellular contacts and sporadic apoptosis in the odontoblasts of ***Dspp***^−1fs^ mice

**Figure 3:**
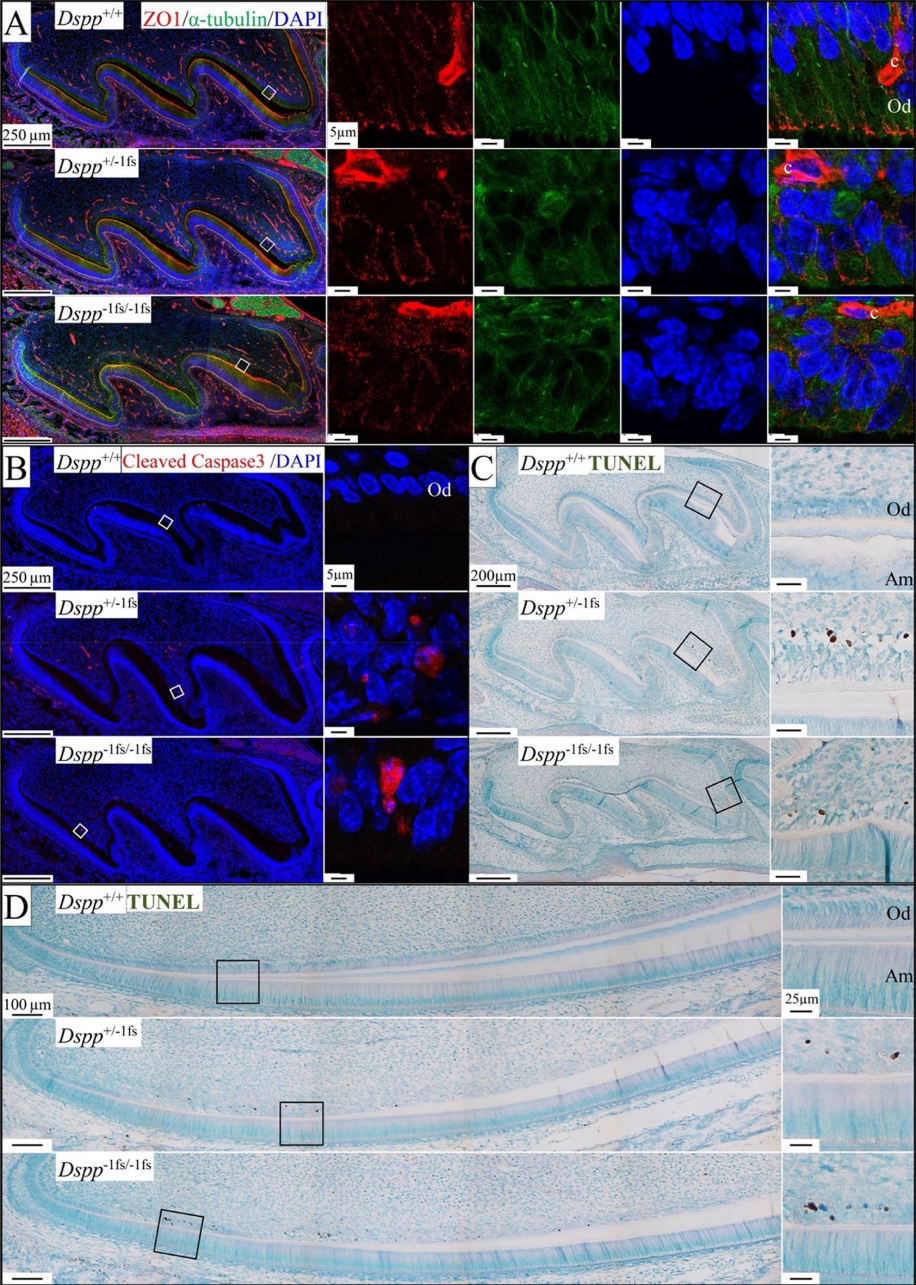
Loss of tight junctions and sporadic apoptosis in the odontoblasts of *Dspp*^−1fs^ mice. Shown are D3 maxillary first molars (A-C) and D14 mandibular incisors (D). (**A**) Immunohistochemistry showing zonula occludens-1 (ZO1) (red; tight junctions), α-tubulin (green; boundary of cells), and DAPI (blue; nucleus). Capillaries (c) show strong ZO1 staining. In *Dspp*^+/+^ odontoblasts, ZO1 signal is observed in focal locations along the lateral plasma membrane, most prominently at the distal tight junctions. In *Dspp*^−1fs^ mice, odontoblasts displayed irregular and diffuse ZO1 signals. (**B**) Immunohistochemistry of cleaved caspase 3 shows cleaved caspase 3 only in *Dspp*^−1fs^ (and not in *Dspp*^+/+^) odontoblasts. (**C**, **D**) TUNEL assay. Many *Dspp*^−1fs^ odontoblasts are TUNEL-positive (brown). Od—odontoblast; Am—ameloblast.

We used an antibody against ZO1 (zonula occludens 1), a tight junction adaptor protein, together with α-tubulin to map out the boundary of cells, to visualize and compare odontoblast intercellular contacts in *Dspp*^+/+^ (WT) and *Dspp*^−1fs^ mice (Fig. 3A). In WT odontoblasts, ZO1 signal was observed in clusters along the lateral plasma membrane, but most prominently at the distal junctions. *Dspp*^−1fs^ odontoblasts lacked their regular organization of tight junctions. Some odontoblasts displayed ZO1 signal on all sides of the plasma membrane, while others showed only sporadic signal on the membrane. The loss of organized tight junctions in the odontoblasts explained the loss of polarity and morphological changes in *Dspp*^−1fs^ odontoblasts.

After observing characteristics of apoptosis in odontoblasts using FIB-SEM, we further tested for apoptosis using multiple approaches. The activation (cleavage) of caspase 3, an executioner caspase, can be triggered by both intrinsic and extrinsic apoptosis^29^. An antibody targeting cleaved caspase 3 detected apoptosis in some *Dspp*^−1fs^ odontoblasts (Fig. 3B). The TUNEL assay detects DNA fragmentation, the last phase of apoptosis. TUNEL-positive odontoblasts were also observed in the *Dspp*^−1fs^ mice (Fig. 3C,D). Together, these observations demonstrated that apoptosis occurs, but suggested it is too limited to account for the extensive pathosis evident in *Dspp*^−1fs^ odontoblasts.

### Intracellular accumulation and endoplasmic reticulum (ER) retention of DSPP in the odontoblasts and ameloblasts of ***Dspp***^P19L^ and ***Dspp***^−1fs^ mice

**Figure 4:**
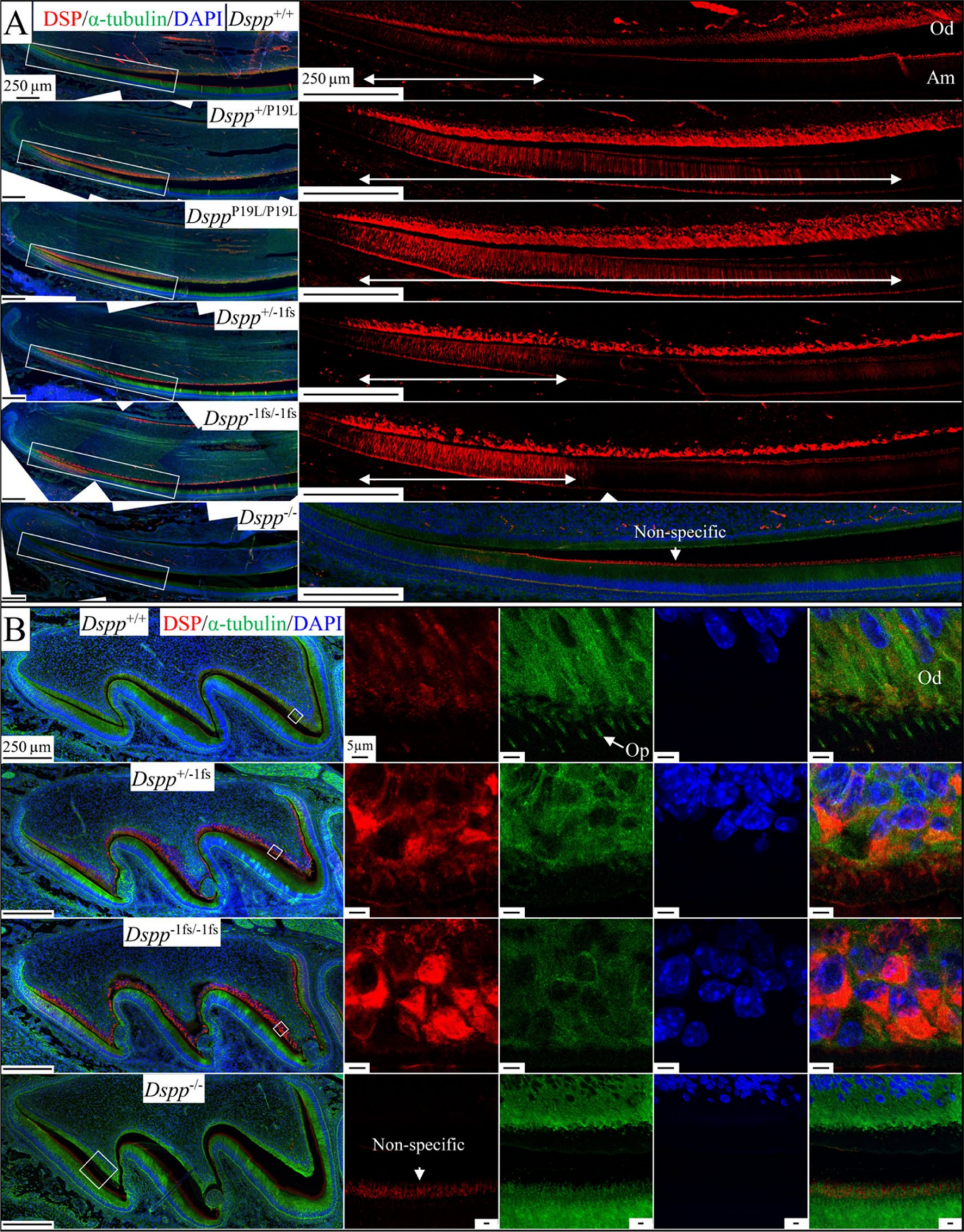
Mutant DSPP accumulation within the odontoblasts and ameloblasts of *Dspp*^P19L^ and *Dspp*^-1fs^ mice. Immunohistochemistry on D14 mandibular incisors (A) and D3 maxillary 1st molars (B), showed DSP (red), α-tubulin (green; boundaries of cells), and DAPI (blue; nucleus). The anti-DSP antibody showed some light, non-specific signal associated with the ameloblast Tomes’ processes in the *Dspp*^−/−^ mice. (**A**) In *Dspp*^+/+^ incisors, intracellular DSP signal intensity was low to medium in odontoblasts (Od) and low intensity in ameloblasts (Am). Strong intracellular accumulation of DSPP is observed in odontoblasts and ameloblasts of *Dspp*^P19L^ and *Dspp*^−1fs^ mice. White double arrows mark ameloblasts with detectable intracellular DSP signal, indicating *Dspp*^P19L^ ameloblasts with prolonged retention of DSPP. Scale bar: 250 μm. (**B**) In *Dspp*^−1fs^ maxillary 1st molars, intracellular DSP signals show that DSPP is seen in odontoblasts and neighboring pulp cells. The DSP-positive cells are not tall columnar in shape, arranged in a sheet, or extending odontoblastic processes (Op) into dentin. Scale bars: 250 μm (left)/5 μm (right). See also Figure S3A.

We previously reported the intracellular accumulation of DSPP in the odontoblasts of *Dspp*^P19L^ and *Dspp*^−1fs^ mice^12,16^. Here, we used immunohistochemistry to compare the intracellular accumulation of DSPP in odontoblasts and ameloblasts in D14 mandibular incisors from WT, *Dspp*^P19L^, *Dspp*^−1fs^ and *Dspp*^−/−^ mice (Fig. 4A). In the WT, intracellular DSP signal was detected at medium intensity in odontoblasts and low intensity in ameloblasts. In both *Dspp*^P19L^ and *Dspp*^−1fs^ incisors, intracellular DSPP signal was significantly elevated in both odontoblasts and ameloblasts and extended further incisally in ameloblasts relative to the WT. The DSP signal in *Dspp*^−1fs^ odontoblasts appeared more concentrated in spots, while the DSP signal in *Dspp*^P19L^ odontoblasts was spread out and occupied almost the whole cytoplasm. The patterns of accumulated DSPP signal highlighted the continuity of the tall sheet of columnar odontoblasts in *Dspp*^P19L^ incisors and its disruption in *Dspp*^−1fs^ incisors. Retention of mutant DSPP within ameloblasts beyond the presecretory stage was only observed in *Dspp*^P19L^ incisors and suggested a mechanism whereby ameloblasts no longer synthesizing DSPP might still be affected by its retention.

DSPP immunohistochemistry of WT and *Dspp*^−1fs^ D3 maxillary first molars using antibodies against α-tubulin clearly showed that the odontoblastic processes, so characteristic of WT odontoblasts, were completely absent in *Dspp*^−1fs^ molars (Fig. 4B, green), whereas the DSPP-positive cells were low columnar cells on the pulp surface, usually polarized, with a large nucleus to cytoplasm ratio and no odontoblastic process (Fig. 4B, red). These findings were confirmed in 3-day-old maxillary 1st molars using an anti-FLAG antibody specific for the DYKDDDDK flag-tag added to the *Dspp*^−1fs^ construct (Figure S3A).

**Figure 5:**
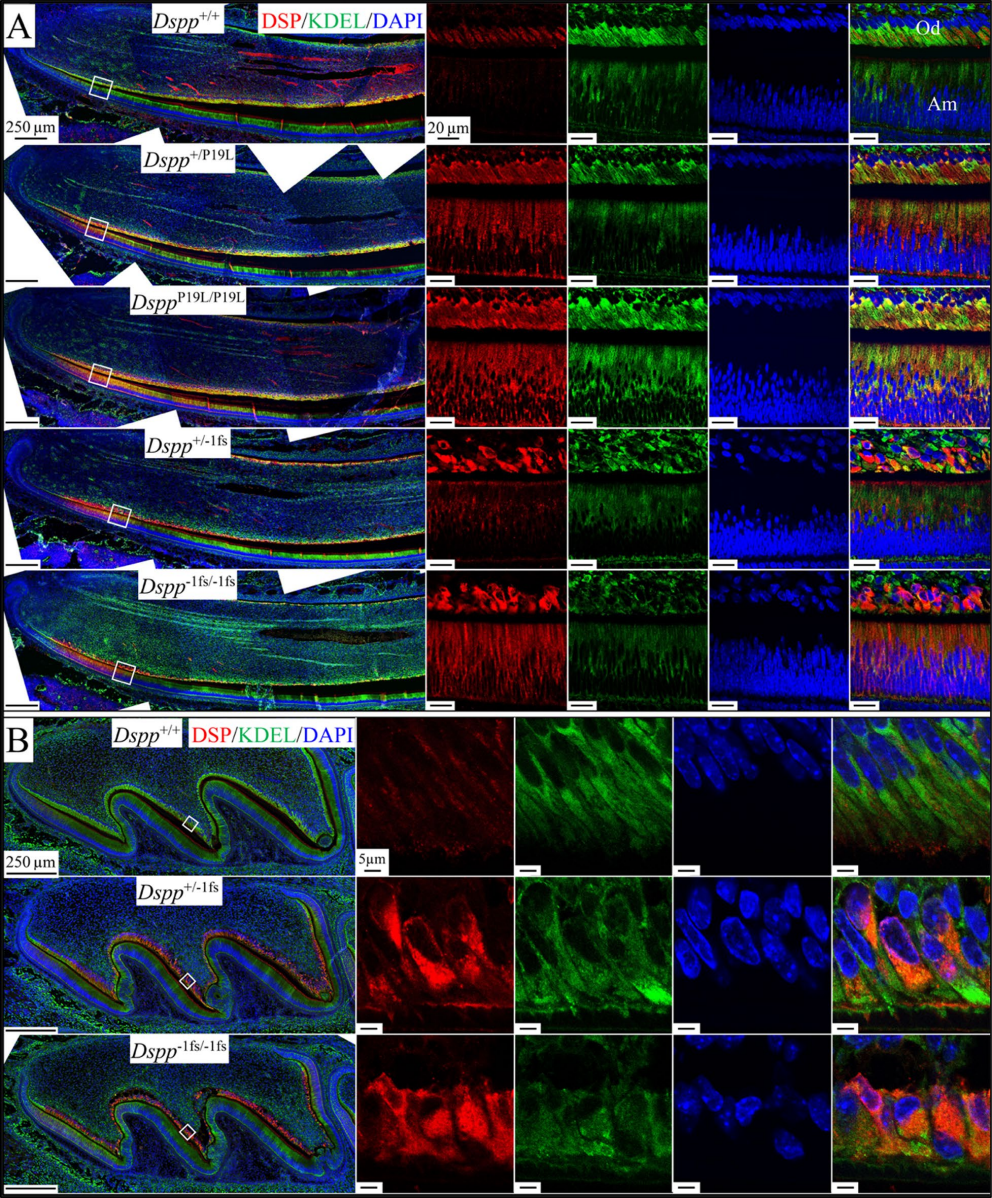
Endoplasmic reticulum (ER) retention of DSPP within the odontoblasts and ameloblasts of *Dspp*^P19L^ and *Dspp*^−1fs^ mice. Immunohistochemistry on D14 mandibular incisors (A) and D3 maxillary 1st molars (B), showing DSP (red), KDEL (green; ER), and DAPI (blue; nucleus). Scale bars: 250 µm (left)/20 μm (right). (**A**) In *Dspp*^P19L^ incisors, DSP and KDEL signals in odontoblasts and presecretory ameloblasts overlap, indicating moderate retention of the p.P19L DSPP protein in the ER. In *Dspp*^−1fs^ incisor odontoblasts, the intracellular DSP predominates over the KDEL signal indicating very high accumulation of the -1 frameshifted DSPP in the ER. Od, odontoblast; Am, ameloblast. (**B**) In *Dspp*^+/+^ molar odontoblasts, KDEL signal is distributed throughout cytoplasm at medium intensity with minimal overlapping DSP signal. In *Dspp*^−1fs^ molar odontoblasts, the DSP and KDEL signals overlap indicating retention of the -1 frameshifted DSPP protein in the ER, whereas there is minimal accumulation of the -1 frameshifted DSPP protein in presecretory ameloblasts. See also Figure S3B.

We investigated the subcellular localization of DSPP accumulations in *Dspp*^P19L^ and *Dspp*^−1fs^ mice using an anti-KDEL antibody (Figs. 5 and S3B). A tetrapeptide motif Lys-Asp-Glu-Leu (KDEL) is an endoplasmic reticulum (ER) retrieval signal at the C-terminal of ER chaperone proteins^30^; thus, this antibody is an ER marker. In WT odontoblasts and ameloblasts minimal overlap was observed between the DSP and KDEL signals in both incisors (Fig. 5A) and molars (Fig. 5B), indicating that the passage of WT-DSPP through the ER was relatively brief and WT-DSPP was efficiently synthesized and secreted by both odontoblasts and presecretory ameloblasts. The DSP and KDEL signals in *Dspp*^P19L^ mice overlapped, with similar levels of overlap exhibited by odontoblasts and presecretory ameloblasts (Fig. 5A). The DSP and KDEL signals in *Dspp*^−1fs^ mice also overlapped, but the DSP strongly predominated over the KDEL signal within *Dspp*^−1fs^ odontoblasts indicating abundant accumulation of the -1 frameshifted DSPP protein in the ER. The DSP and KDEL signals in presecretory ameloblasts also overlapped in *Dspp*^−1fs^ incisors, but the strength of the DSP signal did not predominate. These findings demonstrate that the WT DSPP protein does not accumulate in the ER, whereas the -1 frameshifted DSPP protein accumulates in the odontoblast ER to a much greater extent than does the p.P19L DSPP protein.

### Intracellular retention of DMP1 and Type I Collagen in the odontoblasts of ***Dspp***^−1fs^ mice

**Figure 6:**
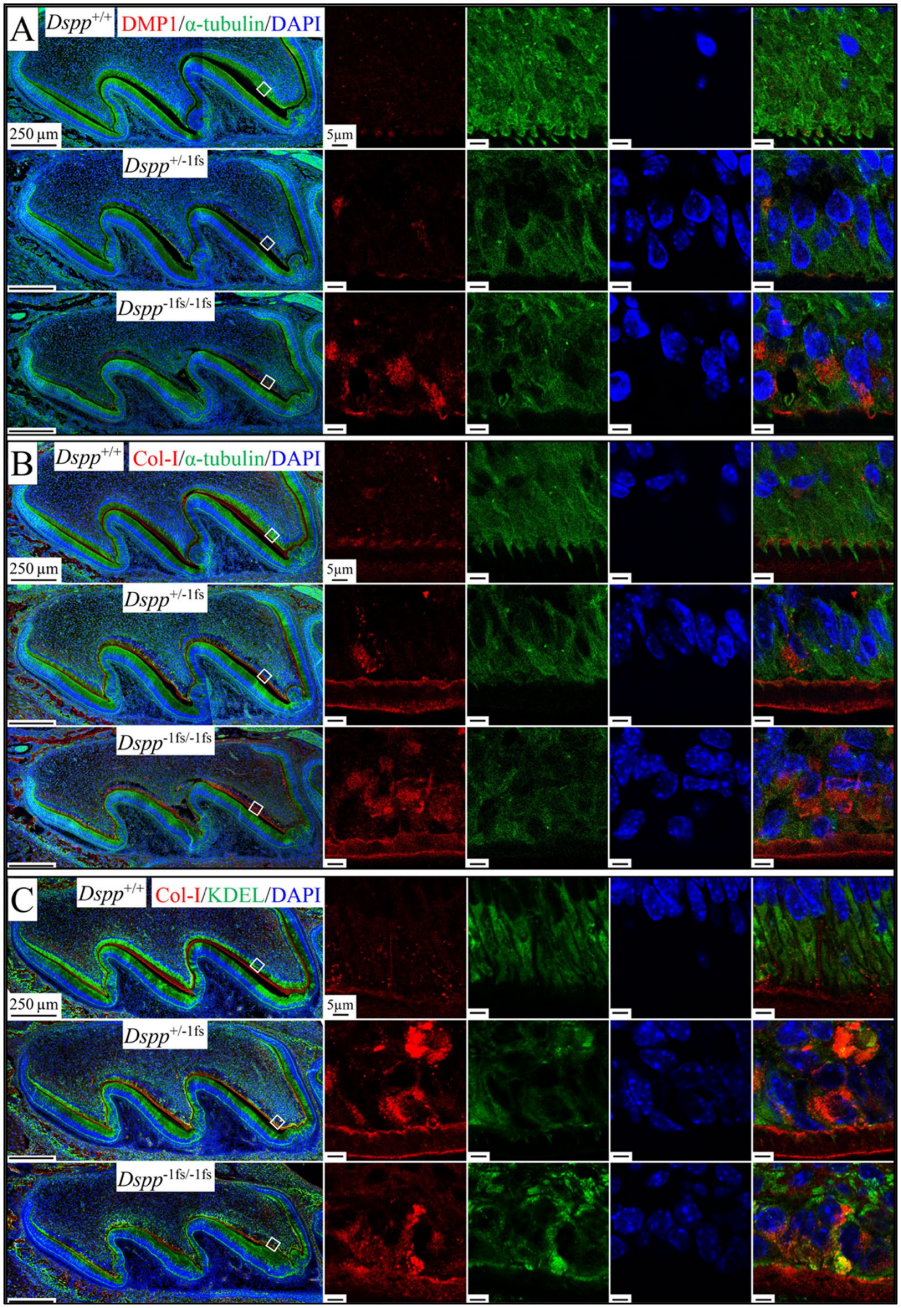
Intracellular retention of DMP1 and type I collagen in the odontoblasts of *Dspp*^−1fs^ mice. Immunohistochemistry on D3 maxillary 1st molars, showing DMP1 (red in A) or type I collagen (red in B&C), α-tubulin (green in A&B; boundary of cells) or KDEL (green in C; ER), and DAPI (blue; nucleus). (**A**) In *Dspp*^+/+^ odontoblasts, intracellular DMP1 is weakly detected at distal end of odontoblast cell bodies. In *Dspp*^-1fs^ odontoblasts, intracellular DMP1 signals are upregulated. (**B**) In *Dspp*^+/+^, type I collagen is most prominent in dentin matrix. In *Dspp*^−1fs^, type I collagen is evident in dentin matrix and within odontoblasts. (**C**) In *Dspp*^+/+^ odontoblasts, most type I collagen locate outside of ER. In *Dspp*^−1fs^ odontoblasts, most intracellular type I collagen signals overlap with KDEL signals. Scale bars: 250 μm (left)/5 μm (right).

Since the -1 frameshifted DSPP protein was retained in the ER, we characterized the expression of DMP1 and type I collagen to see if their expression/secretion had been altered by *Dspp*^−1fs^ expression/retention. In WT odontoblasts, intracellular DMP1 was only weakly detected at the distal end of odontoblast cell bodies (Fig. 6A). In *Dspp*^−1fs^ odontoblasts, intracellular accumulation of DMP1 was observed (Fig. 6A). In the WT mice, type I collagen was most prominent between odontoblastic processes and in the pre-dentin and dentin matrix (Fig. 6B). In the *Dspp*^−1fs^ mice, type I collagen was observed in the pre-dentin and dentin matrix and inside odontoblasts (Fig. 6B). Using the anti-KDEL antibody, we found that most of the type I collagen localized outside of the ER in WT odontoblasts (Fig. 6C). In comparison, most of the intracellular type I collagen signal overlapped the KDEL signal in *Dspp*^−1fs^ odontoblasts (Fig. 6C), indicating the ER retention of type I collagen. The secretions of both DMP1 and type I collagen were altered in *Dspp*^−1fs^ odontoblasts.

### Strong activation of ubiquitin activity in the odontoblasts of ***Dspp***^−1fs^ mice

**Figure 7:**
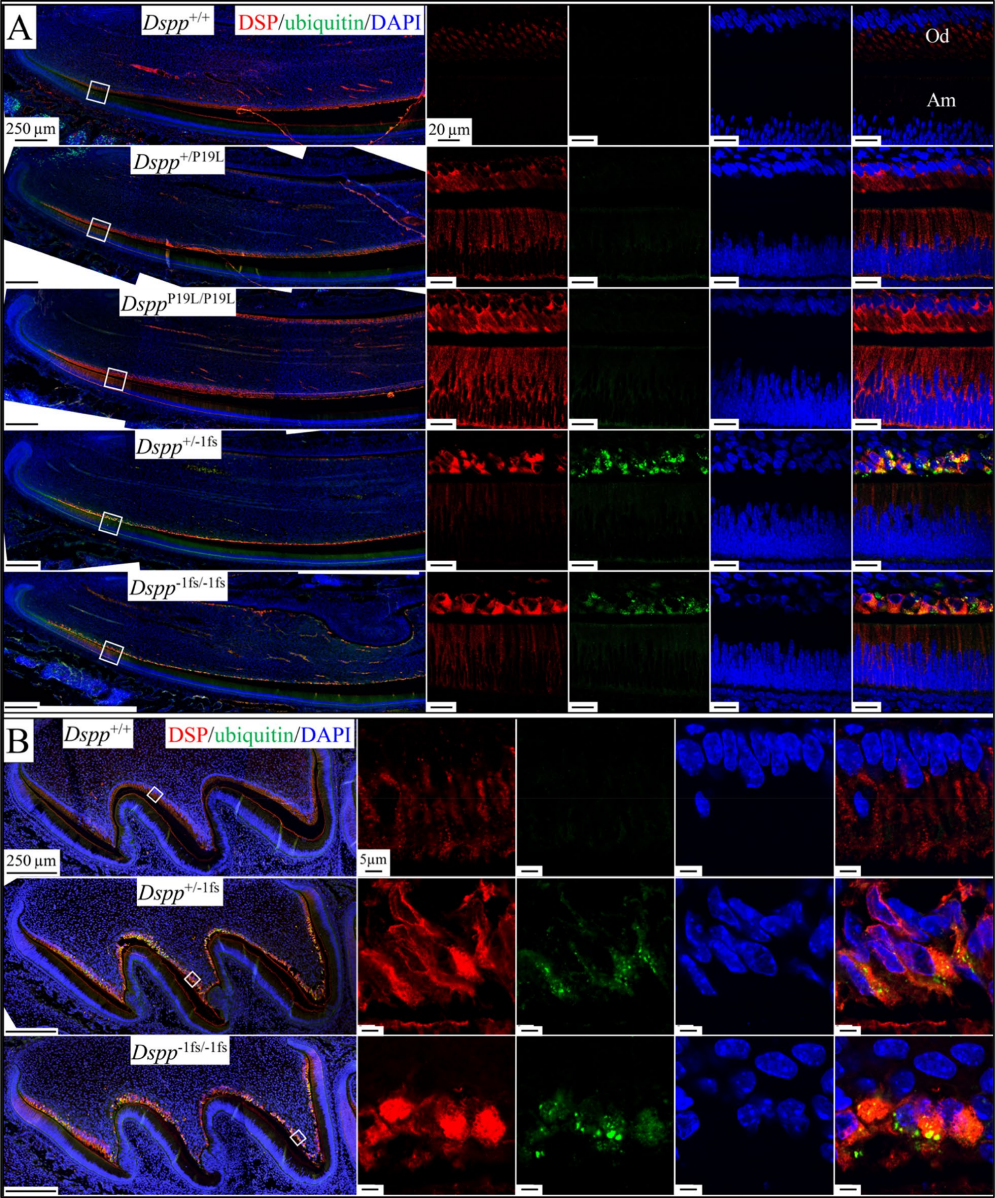
Activated ubiquitin activity in the odontoblasts of *Dspp*^−1fs^ mice. Immunohistochemistry on D14 mandibular incisors (**A**) and D3 maxillary 1st molars (**B**), showing DSP (red), ubiquitin (green), and DAPI (blue; nucleus). Strong ubiquitin signal that partially overlapped the DSP signal was specifically detected in *Dspp*^−1fs^ incisor (**A**) and molar (**B**) odontoblasts (Od). Ubiquitin signal was trace or not detected in odontoblasts or ameloblasts (Am) in *Dspp*^+/+^ and *Dspp*^P19L^ incisors (**A**) or in *Dspp*^−1fs^ ameloblasts (**B**). Scale bars: 250 μm (left)/5 μm (right).

Odontoblasts and ameloblasts in *Dspp*^P19L^ and *Dspp*^−1fs^ mice may activate degradative pathways to cope with the intracellular/ER accumulated DSPP proteins. Ubiquitin is a molecular tag common for three degradative pathways: endocytosis, proteasome, and autophagy^24^. We tested for ubiquitin activity in the odontoblasts and ameloblasts of *Dspp*^P19L^ and *Dspp*^−1fs^ mice (Fig. 7). Ubiquitin was barely detectable in the odontoblasts and ameloblasts of WT and *Dspp*^P19L^ mice, as well as the ameloblasts of *Dspp*^−1fs^ mice (Fig. 7A). In contrast, ubiquitin signal was strong in *Dspp*^−1fs^ odontoblasts, mostly overlapping with DSP signal (Fig. 7A,B), indicating that ubiquitin tagged the -1 frameshifted DSPP protein in *Dspp*^−1fs^ odontoblasts.

### Autophagy activation in the odontoblasts of ***Dspp***^−1fs^ mice

**Figure 8:**
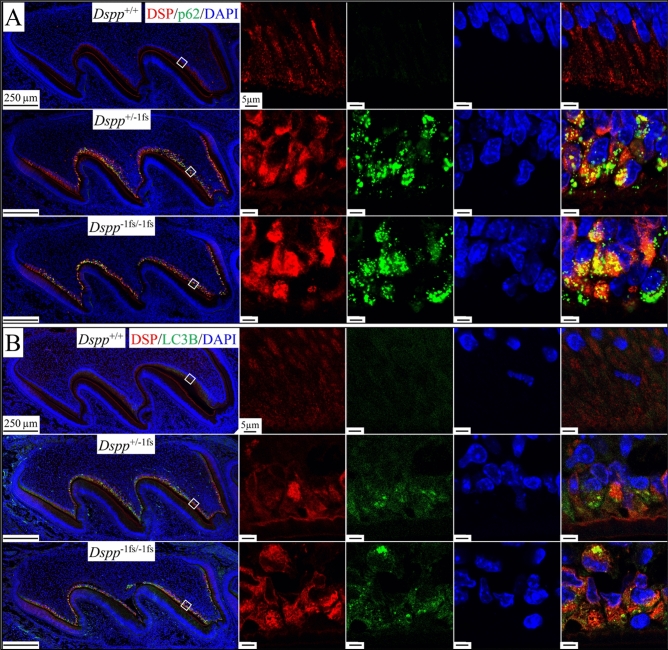
Autophagy is activated in the *Dspp*^−1fs^ odontoblasts. Immunohistochemistry of D3 maxillary 1st molars, showing DSP (red), p62 (green in A; autophagy adaptor) or LC3B (green in B; autophagic vacuoles), and DAPI (blue; nucleus). (**A**) p62 signal was negative in *Dspp*^+/+^ odontoblasts and ameloblasts and in *Dspp*^−1fs^ ameloblasts. Strong p62 signal was detected in *Dspp*^−1fs^ odontoblasts. Most p62 signal colocalized with intracellular DSP, especially where DSP signal was at its highest. (**B**) LC3B signal was detected in *Dspp*^+/+^ odontoblasts at basal levels but were dramatically elevated in *Dspp*^−1fs^ odontoblasts. Some LC3B signal colocalized with intracellular DSP signal. Scale bars: 250 μm (left)/5 μm (right). See also Figure S4.

Since typical autophagic vacuoles were observed in the odontoblasts of *Dspp*^−1fs^ mice using FIB-SEM (Fig. 2), we assessed autophagy activities molecularly. Sequestosome 1 (p62) is an autophagy adaptor that binds to ubiquitylated substrates and LC3 (microtubule associated protein 1 light chain 3) on the membranes of autophagic vacuoles^31^, allowing autophagy substrates to be further processed by the autophagy-lysosome system. Like ubiquitin, the p62 signal was barely detectable in odontoblasts and ameloblasts of WT and *Dspp*^P19L^ mice, and ameloblasts of *Dspp*^−1fs^ mice (Fig. 8A). In contrast, p62 signal was strong in the *Dspp*^−1fs^ odontoblasts (Figs. 8A, S4A). Most of the p62 signal co-localized with DSPP where the intracellular DSPP signal was highest, indicating that autophagy recognized by p62 was activated when DSPP was concentrated or aggregated in *Dspp*^−1fs^ odontoblasts.

Next, we tested for autophagic vacuoles using an anti-LC3B antibody (Figs. 8B, S4B). LC3B was expressed at a basal level in WT odontoblasts. However, LC3B was strongly activated in *Dspp*^−1fs^ odontoblasts, and some of its signal co-localized with DSPP. Mature autophagic vacuoles fuse with lysosomes for protein degradation^32^. Lysosomes, as shown by an anti-LAMP1 antibody, were isolated vesicles distributed throughout the distal portion of WT odontoblasts (Figure S5). LAMP1 signal showed minimal overlap with DSP signal in odontoblasts and ameloblasts of WT and *Dspp*^P19L^ mice, as well as the ameloblasts of *Dspp*^−1fs^ mice (Figure S5A). In contrast, enlarged lysosomes at a higher intensity were observed in the *Dspp*^−1fs^ odontoblasts (Figure S5). An overlap of LAMP1 and DSP signals were observed. Sometimes, the lysosome signal was high where DSP signal was weak, presumably showing partially degraded DSPP in autolysosomes. Therefore, both the ultrastructural and molecular findings strongly support the presence of autophagy activation in *Dspp*^−1fs^ odontoblasts, but not in *Dspp*^−1fs^ ameloblasts or in the odontoblasts and ameloblasts of WT and *Dspp*^P19L^ mice.

### ER-phagy in the odontoblasts of ***Dspp***^−1fs^ mice

**Figure 9:**
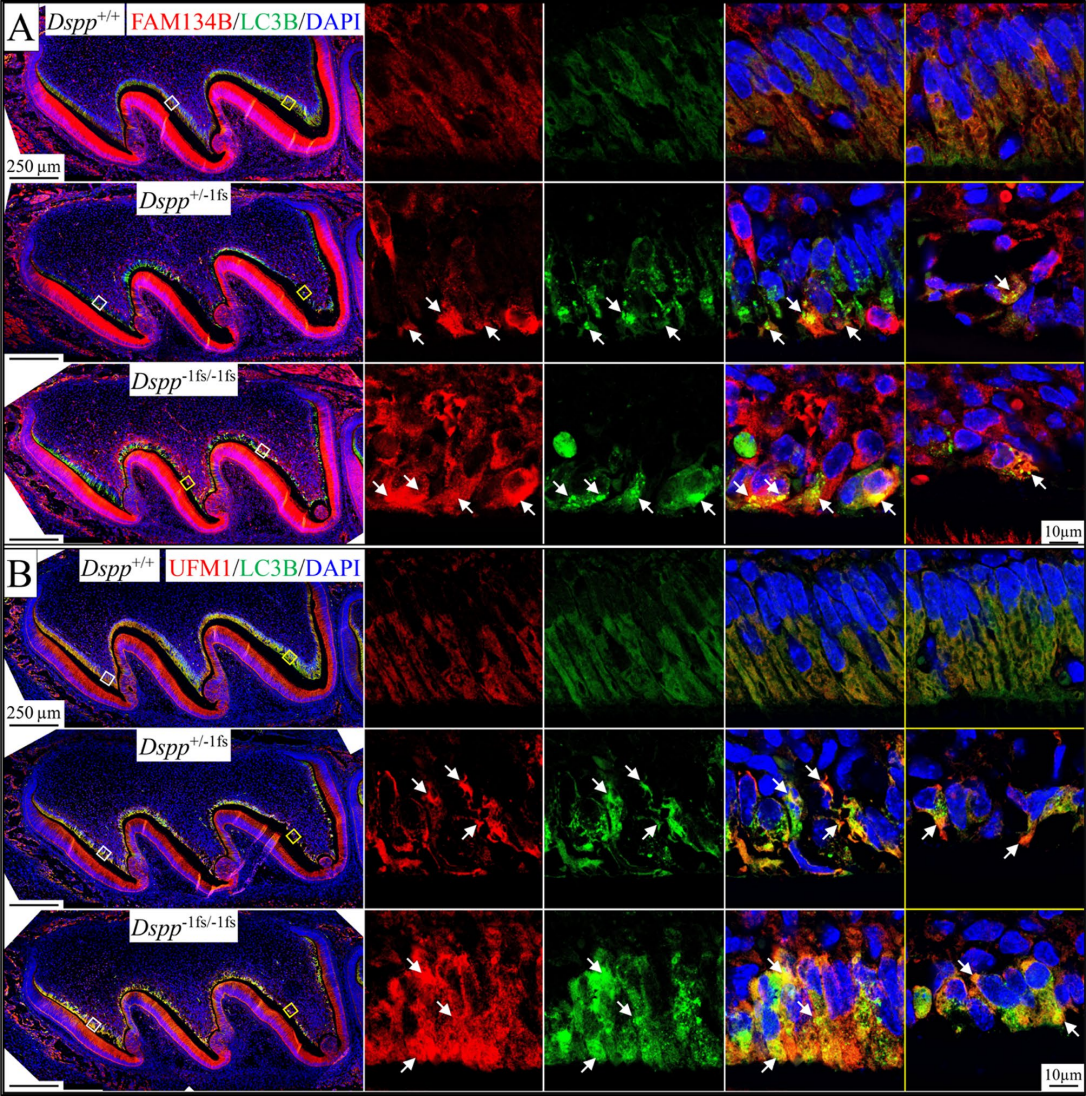
ER-phagy activity in the *Dspp*^−1fs^ odontoblasts. Immunohistochemistry on the D3 maxillary 1st molars, showing FAM134B (red; ER-phagy adaptor) or UFM1 (red; UFMylation), LC3B (green; autophagic vacuoles), and DAPI (blue; nucleus). Two box areas (with white and yellow outlines) are shown at higher magnification on the right. Individual signals from each antibody and merged signals are shown for white outline areas. Only merged signals are shown for yellow outline areas. (**A**) FAM134B signal distributed throughout the cytoplasm of *Dspp*^+/+^ odontoblasts. FAM134B signal congregated regionally in *Dspp*^−1fs^ odontoblasts, partially overlapping the LC3B signal (arrows). (**B**) UFM1 signal was detected in odontoblasts, ameloblasts, and bone cells. UFM1 signal was distributed evenly throughout *Dspp*^+/+^ odontoblast cytoplasm. In *Dspp*^−1fs^ odontoblasts, UFM1 signal congregated regionally, partially overlapping the LC3B signal (arrows). Scale bars: 250 μm (left)/10 μm (right).

FAM134B (family with sequence similarity 134 member B; also called RETREG1, reticulophagy regulator 1) is an ER-phagy adaptor^27^. *Fam134b* mRNA was detected in odontoblasts at a lower level compared to ameloblasts (Figure S6). *Fam134b* expression level was similar between WT and *Dspp*^−1fs^ odontoblasts (Figure S6). FAM134B protein was detected throughout the cytoplasm of WT odontoblasts (Fig. 9A). In *Dspp*^−1fs^ odontoblasts, FAM134B signal concentrated regionally, partially co-localizing with LC3B signal (Fig. 9A). We also analyzed the distribution of UFM1 (ubiquitin-fold modifier 1), a ubiquitin-like protein that post-translationally modifies targeted proteins for UFMylation associated with ER-phagy^33,34^. UFM1 signal was detected in odontoblasts, ameloblasts, and bone cells (Fig. 9B). In WT odontoblasts, UFM1 signal was distributed evenly throughout the cytoplasm. In *Dspp*^−1fs^ odontoblasts, UFM1 signal concentrated regionally, partially co-localizing with LC3B (Fig. 9B). These immunostaining findings, together with the FIB-SEM finding that *Dspp*^−1fs^ odontoblasts contain double-membraned vesicles containing fragmented ER (Fig. 2f) and autophagic vacuoles containing remanent organelles (Fig. 2c), confirmed the presence of ER-phagy in the odontoblasts of *Dspp*^−1fs^ mice.

## Discussion

Dentinogenesis imperfecta (DGI) was first documented in 1883^35,36^. With accumulating cases of autosomal dominant inherited dentin disorders, Shields et al. proposed a classification system^11^ for these disorders. The dentin sialophosphoprotein (*DSPP*) gene was characterized in 1997^4^ and determined to be the causative gene for non-syndromic DGI-II, DGI-III and dentin dysplasia type II (DD-II)^5,37,38^, all of which follow an autosomal dominant pattern of inheritance. As a growing number of disease-causing human *DSPP* mutations were reported, they were found to fall overwhelmingly into two categories: (1) 5’ mutations that altered the amino acid sequence near the signal peptide cleavage site/amino-terminus of the secreted protein, and (2) 3’ -1 frameshift mutations^6,7^ in the DPP coding region. A *Dspp* null mouse model was generated in 2003 that displayed a severe dentin phenotype^39^, but the disease phenotype followed an autosomal recessive pattern of inheritance. The loss of only one *Dspp* allele did not result in a dentin phenotype. Recently, *Dspp*^P19L^ and *Dspp*^−1fs^ mouse models^12,16^ analogous to the 5’ and 3’ disease-causing *DSPP* mutations, respectively, were characterized that exhibited dental defects inherited in a dominant pattern and were more analogous to inherited conditions in humans. In this report, we further characterized the pathological mechanisms in *Dspp*^P19L^ and *Dspp*^−1fs^ mice, hoping to bring insights for the development of future clinical interventions.

Human *DSPP* 5’ mutations sometimes affect both dentin and enamel formation in humans, resulting in rapid enamel attrition and increased risk of pulp exposure^6^. These mutations typically affect the first three amino acids (IPV) following the signal peptide cleavage site and alter DSPP trafficking^40^. SURF4, a cargo protein that binds to the IPV motif, prioritizes the exiting of DSPP from ER^41^. In *Dspp*^P19L^ mice, malformations of dentin and enamel were observed, and were associated with the intracellular accumulation of DSPP proteins^12,18^. Here we showed that both odontoblasts and ameloblasts in the *Dspp*^P19L^ mice displayed strong endoplasmic reticulum (ER) retention of DSPP proteins. Although *Dspp*^−1fs^ mice also showed the accumulation of DSPP proteins in the ER, the ER retention of DSPP proteins in the *Dspp*^P19L^ mice was much stronger, as indicated by the number of ameloblasts with detectable intracellular DSPP signal (Fig. 4). Interestingly, strong ER retention did not activate ubiquitin in odontoblasts or ameloblasts of *Dspp*^P19L^ mice, indicating neither ERAD nor autophagy were activated. A plausible pathological mechanism for *Dspp*^P19L^ odontoblasts or ameloblasts seems to be the unfolded protein response (UPR), but further investigations are warranted.

The 3’ *DSPP* mutations, on the other hand, were exclusively -1 frameshift mutations that changed the repetitive hydrophilic/acidic amino acid sequences into repetitive hydrophobic amino acid sequences of various lengths, but always longer than the native protein^6^. Unlike the accumulated DSPP signals that distributed throughout cytoplasm in *Dspp*^P19L^ mice, the intracellular DSPP in *Dspp*^−1fs^ mice concentrated in spots. Considering the high level of *DSPP* expression and predicted hydrophobicity of the frameshifted DSPP protein in *Dspp*^−1fs^ mice, it likely forms protein aggregates within the secretory pathway, which may affect the secretion of DMP1 and type I collagen in the *Dspp*^−1fs^ odontoblasts. *Dspp*^−1fs^ odontoblasts displayed strong ubiquitin activity and autophagy activation by both molecular and ultrastructural analyses. The autophagy activity involved partial degradation of DSPP in the ER, where the hydrophobic frameshifted protein was actively synthesized, causing ER-phagy.

Pathological mechanisms and their resulting phenotypes are multifactorial. Ameloblasts and odontoblasts are derived from dental epithelium and neural crest cells (ecto-mesenchyme), respectively^42,43^. In addition, enamel and dentin mineralize in distinct ways^44–46^. Dentin mineral foci first appear in pre-dentin, and associate with collagen bundles near the overlying sheet of ameloblasts. These mineral foci coalesce into a continuous layer of mineralized dentin, which gradually thickens by continued deposition of dentin mineral on the odontoblast side of dentin. Characteristic enamel mineral ribbons initiate on the tips of mineralized collagen fibers that are closely associated with the distal membrane of secretory ameloblasts and elongate in the direction that the ameloblast membrane retreats, leading to the organized appositional growth of enamel^47^. When enamel reaches full thickness, ameloblasts transition into maturation stage ameloblasts that reabsorb residual enamel matrix proteins and deposit ions on the sides of the enamel crystals that initially formed during the secretory stage^48^. In the presence of abnormal stimuli caused by expression of the mutant DSPP, the secretory activity and cellular responses of ameloblasts and odontoblasts are altered, potentially altering the formation of enamel and dentin. *Dspp* is only transiently expressed by presecretory ameloblasts prior to the onset of mineralization but is prominently and continuously expressed by odontoblasts throughout dentin mineralization. To alter enamel formation, there must be a residual effect of transient mutated DSPP expression on ameloblasts no longer expressing DSPP. Not all ameloblasts appear to become pathologically affected following their transient expression of mutated DSPP, causing the enamel malformations to be localized.

The cellular responses and their effects on biomineralization were distinct in *Dspp*^P19L^ and *Dspp*^−1fs^ mice. No signs of odontoblast cell death were observed in the *Dspp*^P19L^ mice. *Dspp*^P19L^ odontoblasts retained a significant amount of P19L-DSPP protein within their ER but continued to participate in dentin mineralization. Some of the P19L-DSPP protein was secreted^12^. Since the DSP and DPP sequences in the P19L-DSPP were minimally altered, the P19L-DSPP protein may perform its physiological activities when properly secreted. The *Dspp*^P19L^ dentin and enamel phenotypes arise from reduced secretion of the P19L-DSPP protein and induced pathological changes. In contrast, the − 1fs-DSPP protein has the DPP region replaced with a highly hydrophobic C-terminal sequence that aggregates in both the cell and the pre-dentin matrix and cannot serve the physiological role of DPP. It should be remembered however, that *Dspp*^+/−^ mice deposit normal dentin^39^ and -2 frameshifts in the DPP coding region, which truncate the DPP domain by premature termination^10^, have never been identified as a cause of inherited dentin defects in humans. These observations support the interpretation that a loss of DPP expression from a single DSPP allele does not result in dentin malformations and that the overriding cause of the dental phenotypes in *Dspp*^−1fs^ mice is abnormalities caused by the mutant protein.

Ubiquitin is a common tag for three major protein degradation pathways: ubiquitin–proteasome, autophagy-lysosome, and endo-lysosome systems^24^. The ubiquitin chain linkage type and the resultant three-dimensional structure may determine the selection of degradative pathways^24,49^. The status of substrate proteins may also direct the selection of pathway. The ubiquitin–proteasome system is primarily for short-lived, misfolded, and damaged proteins, while autophagy tends to eliminate large and potentially detrimental cellular components, like protein aggregates and dysfunctional organelles^50,51^. With its tendency for aggregate formation, the strong activation of autophagy in *Dspp*^−1fs^ odontoblasts is not surprising.

Macroautophagy, the most studied type of autophagy (called autophagy thereafter), initiates with the formation of a phagophore, which is often observed in the vicinity of the ER^52^. It remains unclear whether phagophores arise from pre-existing membrane-bounded organelles, such as ER, Golgi apparatus, and mitochondria, or are assembled de novo in the cytosol^52–54^. Following the initiation and elongation of a phagophore, it engulfs ubiquitin-tagged misfolded proteins, protein aggregates, or damaged organelles, and forms a double-membrane-bounded autophagosome^26,53^. Autophagy adaptor proteins, such as p62 and NBR1, bind to ubiquitinated substrates through their UBA (ubiquitin-associated) domain, and to LC3 on the autophagosome membrane through their LIR (LC3-interacting region) domain, fostering selective autophagy^31,55^. The lysosome then fuses with an autophagosome, eliminates the inner autophagosome membrane, and acidifies the compartment environment, leading to the formation of an autolysosome^25^. Within an autolysosome, lysosomal enzymes facilitate the degradation of engulfed cytoplasmic materials^32^. Other than macroautophagy, two other types of autophagy are microautophagy and chaperone-mediated autophagy, both occurring directly on lysosomes^32,56,57^. Atg (autophagy-related) proteins govern the autophagy process^53,58^. For example, LC3 is a member of the Atg8 proteins. In *Dspp*^−1fs^ odontoblasts, we observed single-membrane-bounded degradative autophagic vacuoles containing cytoplasmic materials at different degradative stages more often than double-membrane-bounded autophagic vacuoles. Specifically, we identified a total of 6 double-membrane-bounded autophagic structures from Areas -3 to S4 (annotations in Figure S2A) in *Dspp*^−1fs^ odontoblasts (Figure S7). In comparison, we did not observe any similar structures in the corresponding areas of WT odontoblasts. Strong ubiquitin, p62 and LC3B signals that co-localize with DSPP protein further confirm autophagy activation in the odontoblasts of *Dspp*^−1fs^ mice molecularly.

The selective autophagy of ER, or ER-phagy, referring to the direct engulfing of ER cisternae by autophagosome, is a key ER remodeling process to alleviate ER stress^59^. The ER-phagy adaptors, mostly ER membrane proteins with LIR domain, are the key to this process^27^. FAM134B is the first identified ER-phagy receptor that possesses 2 reticulon domains to modulate ER membrane curvature^60,61^. FAM134B oligomerizes under ER stress to drive ER membrane scission for ER-phagy^62^. No apparent change in *Fam134b* mRNA expression is noted, but FAM134B protein congregates in *Dspp*^−1fs^ odontoblasts, suggesting the oligomerization of FAM134B. Procollagens are abundantly produced and about 20% of newly synthesized type I procollagen is degraded by autophagy-lysosome system due to inefficient folding or secretion^63,64^. Misfolded type I procollagen is recognized by ER chaperone calnexin that interacts with ER-phagy adaptor FAM134B. FAM134B further binds LC3 to be captured by autophagosomes^64^. Odontoblasts produce a collagen-rich pre-dentin matrix; thus, basal levels of LC3B and FAM134B are detected in odontoblasts. In *Dspp*^−1fs^ odontoblasts, the abundant −1fs-DSPP protein in the ER activates ER-phagy (indicated molecularly by LC3B co-localizing with congregated FAM134B) and ultrastructurally by double-membraned autophagic vacuoles engulfing fragmented ER cisternae. ER-phagy requires ER-resident UFMylation, a ubiquitin-like post-translational modification^34^. We detected regionally concentrated UFM1 signal in *Dspp*^−1fs^ odontoblasts, which is associated with ER-phagy activity.

The dentin in *Dspp*^P19L^ and *Dspp*^−1fs^ mice likely represents reactionary and reparative dentin, respectively^16^. The distinction between these two types of tertiary dentin lies in the survival (reactionary) or death (reparative) of the original odontoblasts^65,66^. Cell death is morphologically classified into three types: apoptosis, autophagic cell death, and necrosis^29,67–69^. With the variations of cell death being revealed, the nomenclature of cell death becomes complicated and most molecular details remain obscure^29,70^. Morphologically, apoptosis is featured by cell shrinkage, membrane blebbing, and chromatin condensation^29,69^. Apoptosis occurs by intrinsic and extrinsic pathways and relies on the BCL-2 family of proteins and caspase proteases^69,71^. Characteristic apoptotic odontoblasts were observed sporadically in the *Dspp*^−1fs^ mice. Necrosis is characterized by plasma membrane rupture and disruption of organelle structure^29,69^. Autophagic cell death is featured by the autophagic vacuoles and the engagement of autophagy machinery^69^. Autophagy is mainly pro-survival. It is controversial whether autophagic cell death is merely representative of a failed survival attempt or if autophagy itself can be solely responsible for cell death independent of apoptosis and necrosis^69,72–75^. In the case of *Dspp*^−1fs^ odontoblasts, autophagic activities were detected in most cells whereas apoptotic activities were detected sporadically. Cells from sub-odontoblast layers showed the expression of DSPP and DMP1 (an early odontoblast marker) to perform odontoblast activities. This supports the notion that the original *Dspp*^−1fs^ odontoblasts die, and odontoblast-like cells are recruited to participate in reparative dentin formation. *Dspp*^−1fs^ odontoblast cell death is associated principally with autophagy and sometimes with apoptosis.

With further molecular investigations of DGI using *Dspp*^P19L^ and *Dspp*^−1fs^ mice, we can see a predominant ER retention in the odontoblasts and ameloblasts of the *Dspp*^P19L^ mice, and strongly activated autophagy activities in the odontoblasts of *Dspp*^−1fs^ mice. Better understanding of the molecular details of the odontoblast pathosis are essential for the development of therapeutic interventions for the two types of DGI. For DGI-III due to 5’ *DSPP* mutations, the administration of chemical chaperones may push the secretion of mutant DSPP that maintains a WT DPP sequence. The alleviation of the ER retention of DSPP may reduce the stress in odontoblasts and ameloblasts thus facilitates dentin and enamel formation. The DGI-II caused by 3’ -1 frameshift *DSPP* mutations is unlikely to be remedied, because the − 1fs-DSPP proteins is noxious to odontoblasts and the pathological changes are dramatic. Characterization of the alternative dental phenotypes of *Dspp*^P19L^ and *Dspp*^−1fs^ and characterization of their different pathological mechanisms strongly support the recent revisions made to the Shields classification^6^.

In addition to the mechanistic investigations of DGI, the *Dspp*^−1fs^ mice may serve as a valuable tool for the study of autophagy, ER-phagy, and autophagic cell death. Pathological autophagy activities are localized to odontoblasts, minimizing concerns of adverse effects due to systemic dysregulations. Autophagy activities are high in odontoblasts and enable the investigation of molecular details of autophagic cell death in a mammalian tissue and can be explored further in the *Dspp*^−1fs^ mouse model.

## Methods and materials

### Animal used in this study

All mice used in this study were housed in Association for Assessment and Accreditation of Laboratory Animal Care International (AAALAC)-accredited facilities and were treated humanely according to protocols approved by the University of Michigan and Texas A&M Institutional Animal Care and Use Committees (IACUC) and were carried out in compliance with ARRIVE guidelines. Experimental protocols were designed along University and National Institutes of Health (NIH) guidelines for the humane use of animals.

### Focus ion beamed scanning electronic microscopy (FIB-SEM)

FIB-SEM protocols followed were described previously in detail^45^. Briefly, seven-week-old mice for this study were anesthetized and perfused with 2.5% glutaraldehyde in 0.08 M sodium cacodylate buffer (pH7.3) with 0.05% calcium chloride. Samples were post-fixed in the same fixative for 4–6 h, then changed to 0.1 M sodium cacodylate buffer (pH7.3). The mandibles were washed several times with 0.1 M sodium cacodylate buffer, lipid-stained with 1% reduced osmium tetroxide for 2 h, dehydrated using an acetone gradient, and cured in pure epoxy. Each incisor was cut into 1-mm-thick cross-sectional slices, and those for analysis were glued onto blank epoxy stubs so that the plane of section was parallel to the long axis of the tooth. The area of starting interest for this study was the point marking the start of mineralization of dentin, designated as Area 0. Area 0 and adjacent 100 µm-wide Areas to the apical side of Area 0 (designated as Area -1, -2, -3, -4 moving more apically) and to the incisal side of Area 0 (designated as Area S1, S2, S3, S4 moving more forward into the secretory stage of amelogenesis) were imaged at various magnifications using a FEI Helios Nanolab 660 DualBeam Focused Ion Beam-Scanning Electron Microscope as described previously^45^.

### Histological section preparation

Heads of 3-day-old (D3) wild type (*Dspp*^+/+^), *Dspp*^+/−1fs^ and *Dspp*^−1fs/−1fs^ mice, and mandibles of 14-day-old (D14) *Dspp*^+/+^, *Dspp*^+/−1fs^, *Dspp*^−1fs/−1fs^, *Dspp*^+/P19L^, and *Dspp*^P19L/P19L^ mice were harvested, fixed in 4% PFA in PBS at 4 °C overnight. The samples were decalcified at 4 °C in 4.13% disodium ethylenediaminetetraacetic acid (EDTA, pH 7.4) with agitation for 4 days (D3 samples) or 12 days (D14 samples). The samples were then dehydrated by an ethanol series, cleared by xylene, embedded in paraffin, and sectioned at 5 μm thickness. Sections were obtained from maxillary 1st molars and mandibular incisors and placed on Fisherbrand Tissue Path Superfrost Plus Gold Microscope Slides (Fisher Scientific) for histological analysis.

### Immunohistochemistry

Paraffin sections were heated at 60 °C for 1 h, rehydrated in xylene then an ethanol series. Antigen retrieval was performed in Tris–EDTA buffer (10 mM Tris Base, 1 mM EDTA, 0.05% Tween 20, pH 9.0). The antigen retrieval container was placed in boiling water for 20 min, followed by cooling down at room temperature (RT) for 20 min. Sections were then blocked in 3% bovine serum albumin, 10% normal goat serum, 0.05% Tween 20 in PBS at RT for 2 h, and incubated in an unconjugated rabbit primary antibody in an antibody dilution buffer (10% normal goat serum, 0.05% Tween 20 in PBS) at RT for 1 h, then at 4 °C overnight. The unconjugated primary antibodies used in this study were: (1) a rabbit polyclonal antibody to DSP protein^76^ diluted 1:2000; (2) a rabbit polyclonal antibody to peptide (DYKDDDDK) (Invitrogen, PA1-984B) diluted 1:1000; (3) a rabbit polyclonal antibody to DMP1^77^ diluted 1:400; (4) a rabbit polyclonal antibody to type I Collagen (abcam, ab21286) diluted 1:250; (5) a rabbit monoclonal antibody (5A1E) to Cleaved Caspase-3 (Asp175) (Cell Signaling Technology, #9664) diluted 1:800; (6) a rabbit polyclonal antibody to GRP78/BiP (abcam, ab21685) diluted 1:200; (7) a rabbit monoclonal (E8Y9R) antibody to FAM134B (Cell Signal Technology, #83,414) diluted 1:200; and (8) a rabbit monoclonal [EPR4264(2)] antibody to UFM1 (abcam, ab109305) diluted 1:250. A secondary antibody, Goat anti‐rabbit IgG(H + L) Secondary Antibody, Alexa Fluor Plus 555 (Invitrogen, A32732), was used at a dilution of 1:1000 for incubation of 1 h at RT. Optionally, a conjugated antibody for dual labelling was incubated for 1 h at RT after the secondary antibody. These conjugated antibodies include: (1) a mouse monoclonal antibody to alpha-tubulin with an AF488 conjugate (abcam, ab195887) diluted 1:100; (2) a rabbit monoclonal [EPR12668] antibody to KDEL with an AF488 conjugate (abcam, ab184819) diluted 1:100; (3) a rabbit monoclonal antibody [EPR8830] to ubiquitin with an AF647 conjugate (abcam, ab205468) diluted 1:200; (4) a rabbit monoclonal antibody [EPR4844] to SQSTM1/p62 with an AF647 conjugate (abcam, ab194721) diluted 1:100; (5) a rabbit monoclonal antibody [EPR18709] to LC3B with an AF488 conjugate (abcam, ab225382) diluted 1:100; and (6) a rabbit monoclonal antibody [EPR21026] to LAMP1 with an AF647 conjugate (abcam, ab237307) diluted 1:100. The section was counterstained in 4′,6-Diamidino-2-phenylindole dihydrochloride (DAPI, 1 μg/mL in distilled water; Sigma, D9542) for 2 min, then mounted in Invitrogen ProLong Gold Antifade Mountant with DAPI (Invitrogen, P36935). Images (not including Fig. 9) were taken using a Nikon A1 confocal combined with an inverted Ti-E microscope. Images in Fig. 9 were taken using a Leica STELLARIS 8 confocal microscope with HyD detectors. Both confocal microscopes were at the Imaging Laboratory of the Michigan Diabetes Research Center (Ann Arbor, MI).

### TUNEL assay

TUNEL assay was performed using the TUNEL Assay kit—HRP-DAB (abcam, ab206386) following the manufacturer’s protocol. Images were taken using a Nikon Eclipse TE300 microscope and photographed using a Nikon DXM1200 digital camera as described previously^16^.

### RNAscope in situ hybridization

In situ hybridization was performed using RNAscope™ 2.5 HD assay – Red as previous described^16^. The probe Mm-Retreg1 (Cat #545,851, targeting NM_025459.3, nt 209–1820) was used. Signal detection was conducted for 10 min.

## Supplementary Information

Supplementary Information.

## Data Availability

The mouse strain, 067179-UNC or B6N; SJL-Dsppem1Jpsi/Mmnc is available at the MMRRC-UNC facility.
