# Supplementary material for: Dentin defects caused by a Dspp−1 frameshift mutation are associated with the activation of autophagy
Source: Sci Rep. 2023 Apr 19;13:6393. doi: 10.1038/s41598-023-33362-1 (PMC10115861; doi:10.1038/s41598-023-33362-1)
Supplement: Supplementary file 1 — Supplementary Information. [file 41598_2023_33362_MOESM1_ESM.docx]

**Dentin Defects Caused by a *Dspp*^-1^ Frameshift Mutation Are Associated with the Activation of Autophagy**

Tian Liang^1^*, Charles E. Smith^1,2^, Yuanyuan Hu^1^, Hong Zhang^1^, Chuhua Zhang^1^,

Qian Xu^3^, Yongbo Lu^3^, Ling Qi^4^, Jan C-C. Hu^1^, and James P. Simmer^1^

^1^Department of Biologic and Materials Sciences, University of Michigan School of Dentistry, 1011 North University, Ann Arbor, MI, USA.

^2^Department of Anatomy & Cell Biology, Faculty of Medicine & Health Sciences, McGill University, Montreal, QC, Canada

^3^Department of Biomedical Sciences and Center for Craniofacial Research and Diagnosis, Texas A&M University College of Dentistry, 3302 Gaston Ave., Dallas, TX 75246, USA.

^4^Department of Molecular & Integrative Physiology, Department of Internal Medicine, Division of Metabolism, Endocrinology and Diabetes, University of Michigan Medical School, 1000 Wall St., Ann Arbor, MI 48105, USA.

**List of Supplemental Figures.**

Fig S1. Focused Ion Beam Scanning Electron Microscopy (FIB-SEM) of a 7-week-old Wild-type Incisor Showing Normal Odontoblast Morphology and Dentin Formation.

Fig S2. FIB-SEM montages of a 7-week-old *Dspp*^-1fs/-1fs^ Incisor Showing Odontoblast Pathosis and Defective Dentinogenesis.

Fig S3. Mutant DSPP accumulated within the odontoblasts of *Dspp*^-1fs^ mice.

Fig S4. Autophagy is activated in *Dspp*^-1fs^ odontoblasts but not *Dspp*^-1fs^ ameloblasts or *Dspp*^P19L^ odontoblasts and ameloblasts.

Fig S5. Strong lysosome activities in the *Dspp*^-1fs^ odontoblasts.

Fig S6. Fam134b expression in *Dspp*^-1fs^ odontoblasts.

Fig S7. Double-membrane-bounded autophagic structures in *Dspp*^-1fs^ odontoblasts.


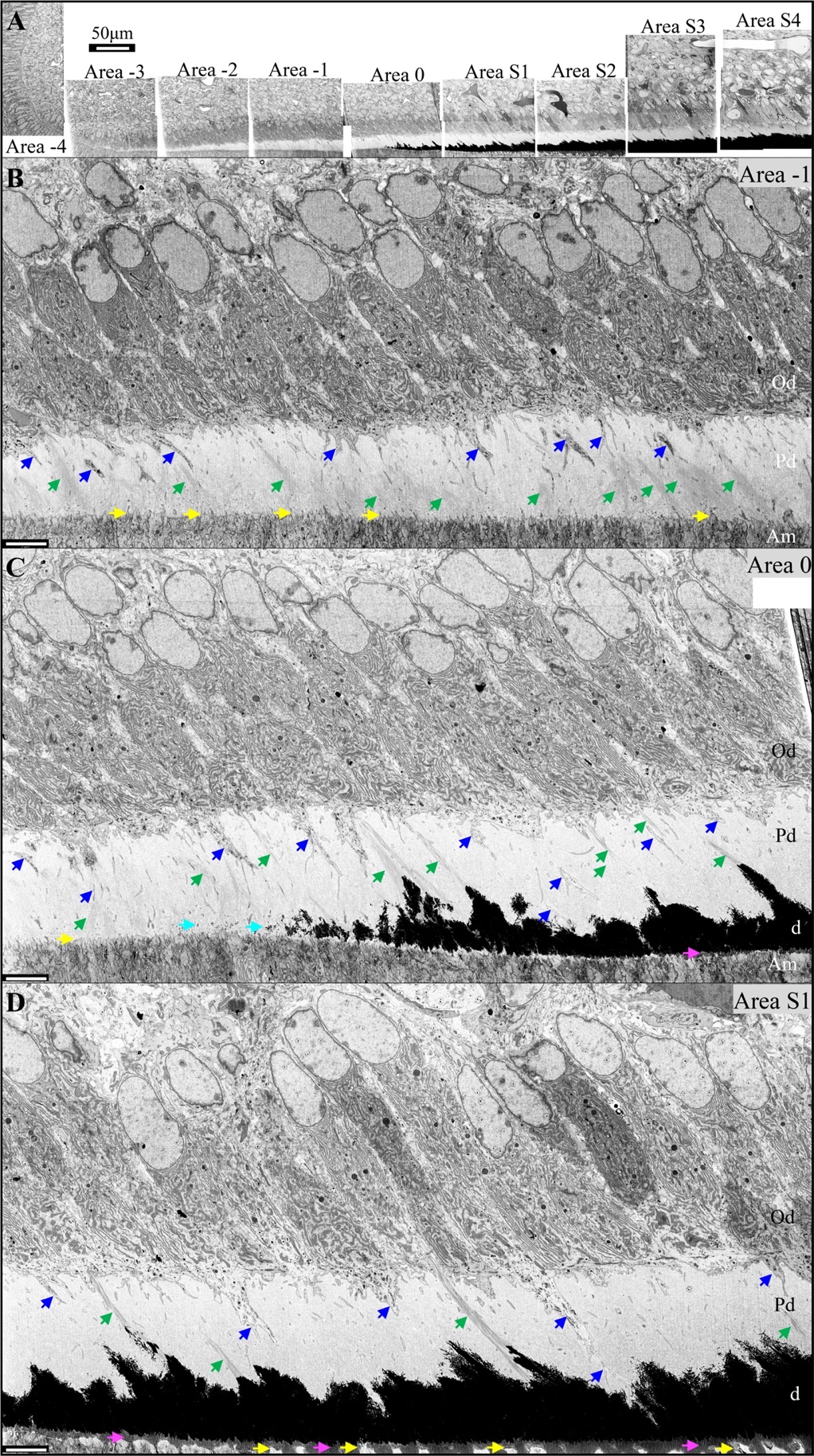


**Fig S1. Focused Ion Beam Scanning Electron Microscopy (FIB-SEM) of a 7-week-old Wild-type Incisor Showing Normal Odontoblast Morphology and Dentin Formation. A.** 2500x FIB-SEM montages extending from the apical loop (left) through the onset of dentin formation (Area 0) and beyond (Area S4). **B.** 5000x FIB-SEM montages of Area -1 showing polarized odontoblasts (Od) with odontoblastic processes (blue arrows) extending in predentin (Pd) containing collagen bundles (green arrows) extending to the irregular distal ends (yellow arrows) of presecretory ameloblasts (Am). **C.** 5000x FIB-SEM montages of Area 0 showing mineral foci (cyan arrows) coalesce into a continuous layer of mineralized dentin (D). Enamel mineral ribbons form near the end of Area 0 (magenta arrow). **D.** 5000x FIB-SEM montage of Area S1. Dentin mineral (d) thickens gradually, whereas predentin (Pd) thickness is relatively constant. Enamel mineral ribbons elongate and ameloblasts develop Tomes’ processes (yellow arrows). Scale bars (B-D): 5 μm.


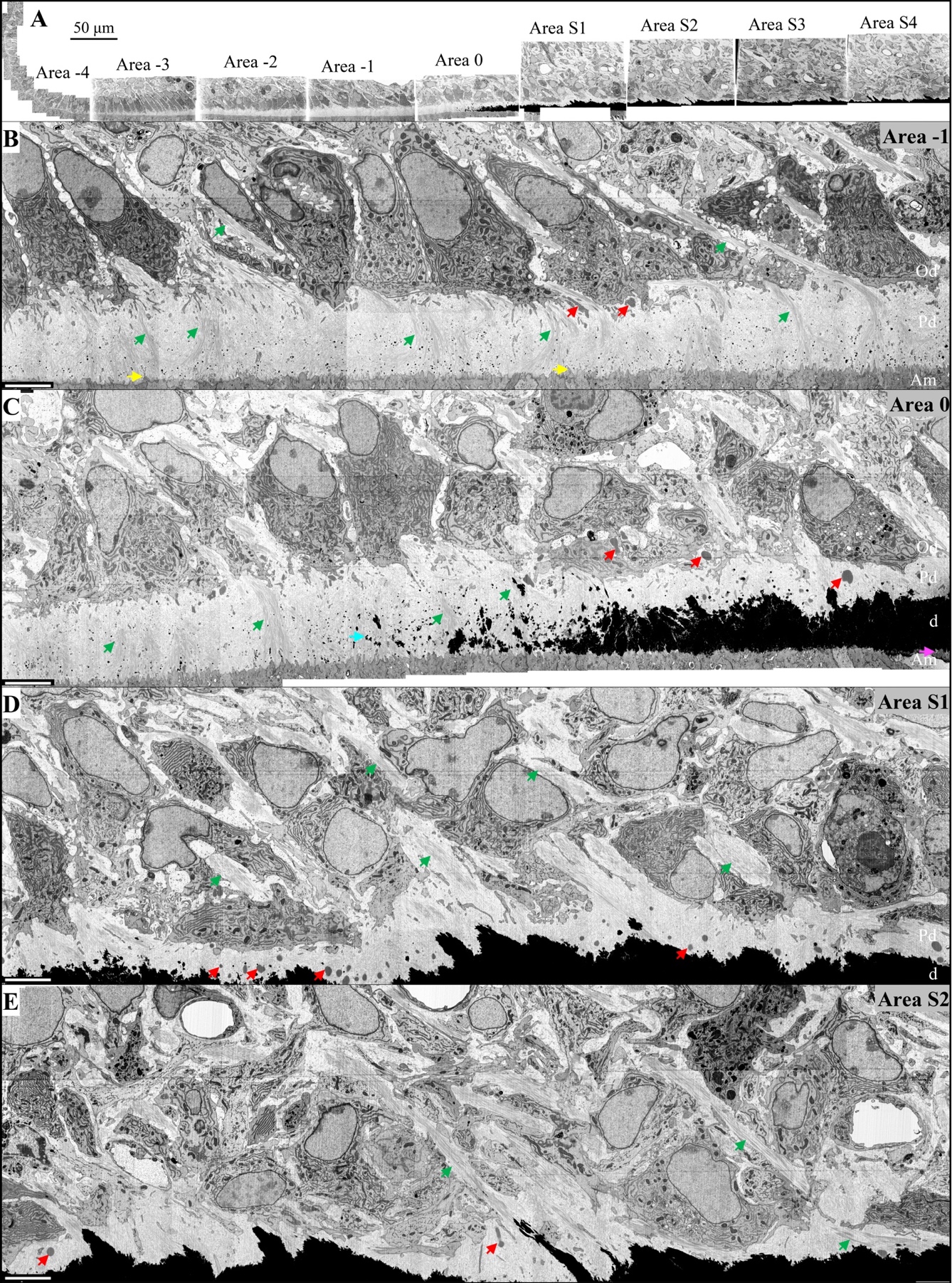


**Fig S2. FIB-SEM montages of a 7-week-old *Dspp*^-1fs/-1fs^ Incisor Showing Odontoblast Pathosis and Defective Dentinogenesis. A**. 2500x FIB-SEM montages extending from the apical loop (left) through the onset of dentin formation and beyond. **B.** 10000x FIB-SEM montages of Area -1. Odontoblasts (Od) remained polarized but were shorter than those in the wild type (Fig S1). Numerous degradative vacuoles were observed in odontoblasts. Intercellular contacts were loose, making collagen bundles (green arrows) between odontoblasts more visible. Extracellular medium-density protein aggregates (red arrows) were observed near pathological odontoblasts. **C.** 10000x FIB-SEM montages of Area 0. Independent mineral foci (cyan arrow) extend over a larger area of predentin (Pd) before they coalesce into a continuous dentin (d) layer. The Pd layer progressively thins as pathological odontoblasts deposit less matrix. Initial enamel ribbons (magenta arrow) appeared near the end of Area 0. **D.** 10000x FIB-SEM of Area S1. The sheet of odontoblasts is apparently replaced by the sub-odontoblastic layer and Pd is thin. **E.** 10000x FIB-SEM montage of Area S2. Nothing resembling an odontoblast layer was observed. Scale bars: 5 μm.


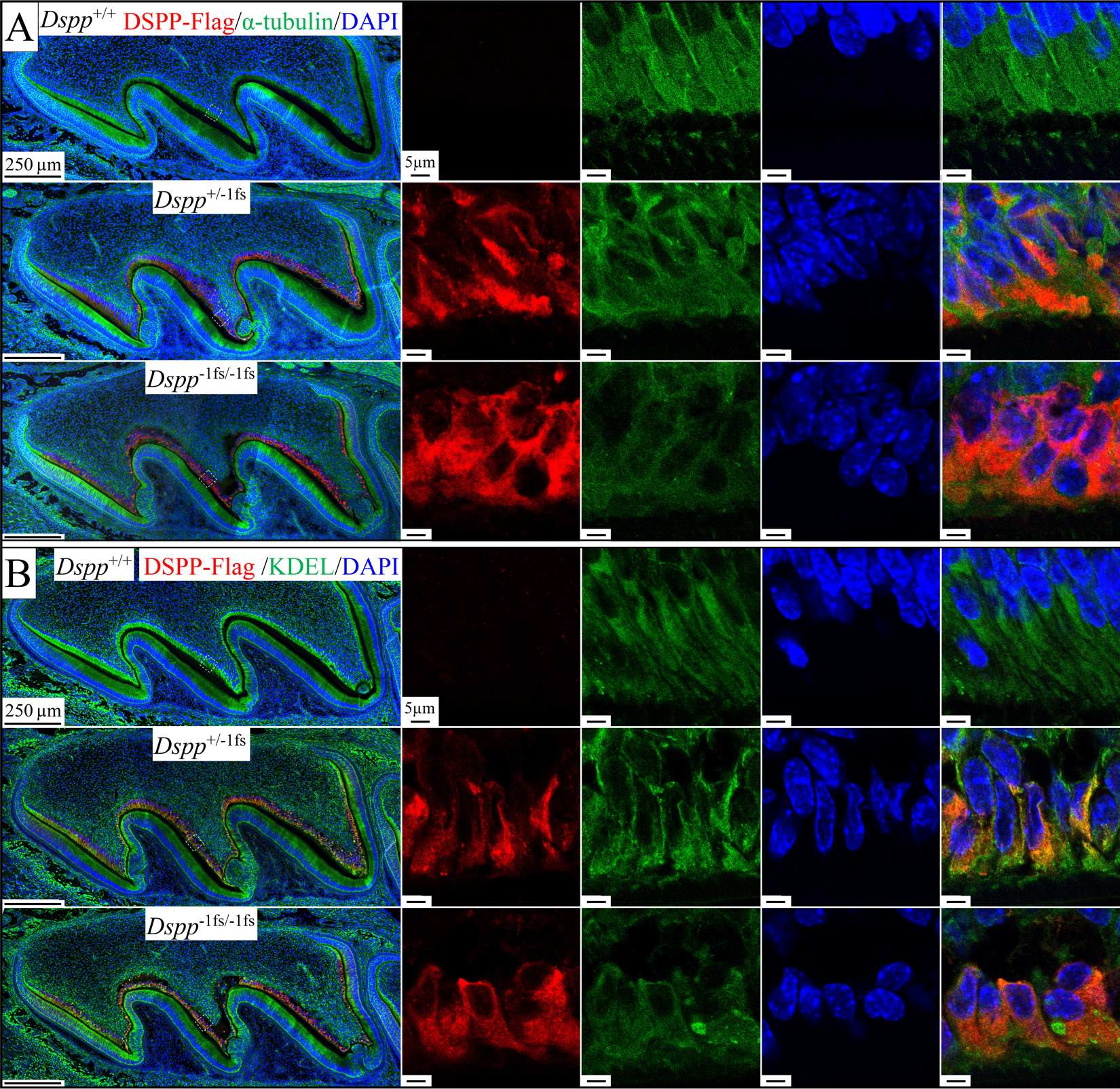


**Fig S3. Mutant DSPP accumulated within the odontoblasts of *Dspp*^-1fs^ mice.** Immunohistochemistry on D3 maxillary 1^st^ molars, showing DSP-Flag (red), α-tubulin (green in A; boundary of cells) or KDEL (green in B; ER), and DAPI (blue; nucleus). A Flag-tag was inserted into DSPP in *Dspp*^-1fs^ mice, so the positive Flag signal localized the -1 frameshifted DSPP specifically. **A.** In *Dspp*^+/+^, no Flag signal is detected. Strong intracellular Flag signal was observed in *Dspp*^-1fs^ odontoblasts. **B**. In *Dspp*^+/+^ odontoblasts, KDEL signal distributed throughout cytoplasm, except odontoblastic processes. In *Dspp*^-1fs^ odontoblasts, KDEL signal was relatively high regionally. Most Flag signal in *Dspp*^-1fs^ odontoblasts overlapped with KDEL signal. Scale bars: 250 μm (left)/5 μm (right).

**
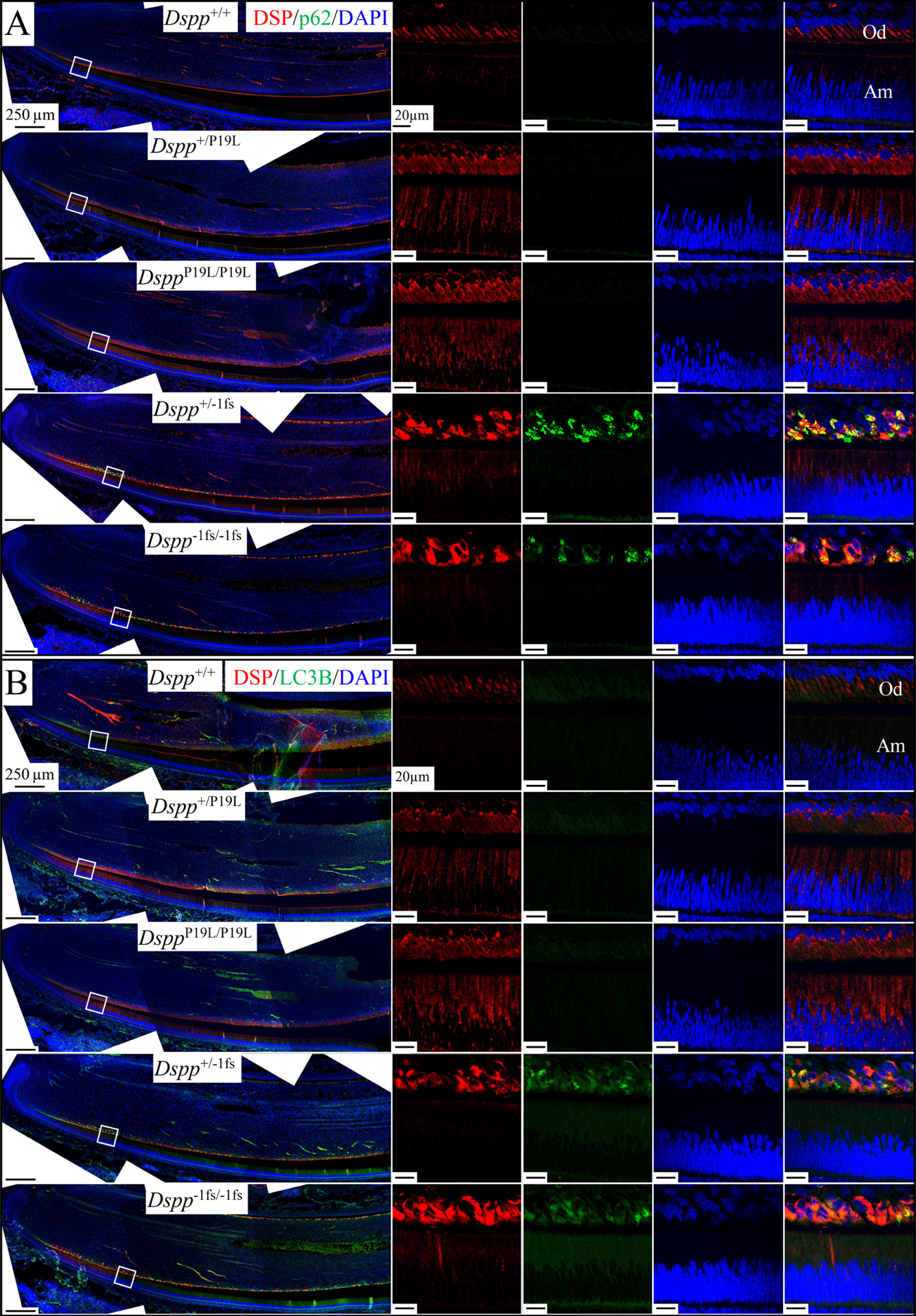
**

**Fig S4.** **Autophagy is activated in *Dspp*^-1fs^ odontoblasts but not *Dspp*^-1fs^ ameloblasts or *Dspp*^P19L^ odontoblasts and ameloblasts.** Immunohistochemistry on D14 *Dspp*^P19L^ and *Dspp*^-1fs^ mice mandibular incisors, showing DSP (red), p62 (green in A; an autophagy adaptor) or LC3B (green in B; autophagic vacuoles), and DAPI (blue; nucleus). **A.** p62 signal was trace in *Dspp*^+/+^ and *Dspp*^P19L^ mice, and in ameloblasts of *Dspp*^-1fs^ mice. p62 signal was strong in *Dspp*^-1fs^ odontoblasts. Most p62 signal colocalized with intracellular DSP signal. **B.** LC3B signal was detected at basal levels in *Dspp*^+/+^ and *Dspp*^P19L^ odontoblasts and was trace ameloblasts of all mice. LC3B signal was dramatically elevated in *Dspp*^-1fs^ odontoblasts. Most LC3B signal colocalized with intracellular DSP signal. Scale bars: 250 μm (left)/5 μm (right). Od, odontoblast; Am, ameloblast.


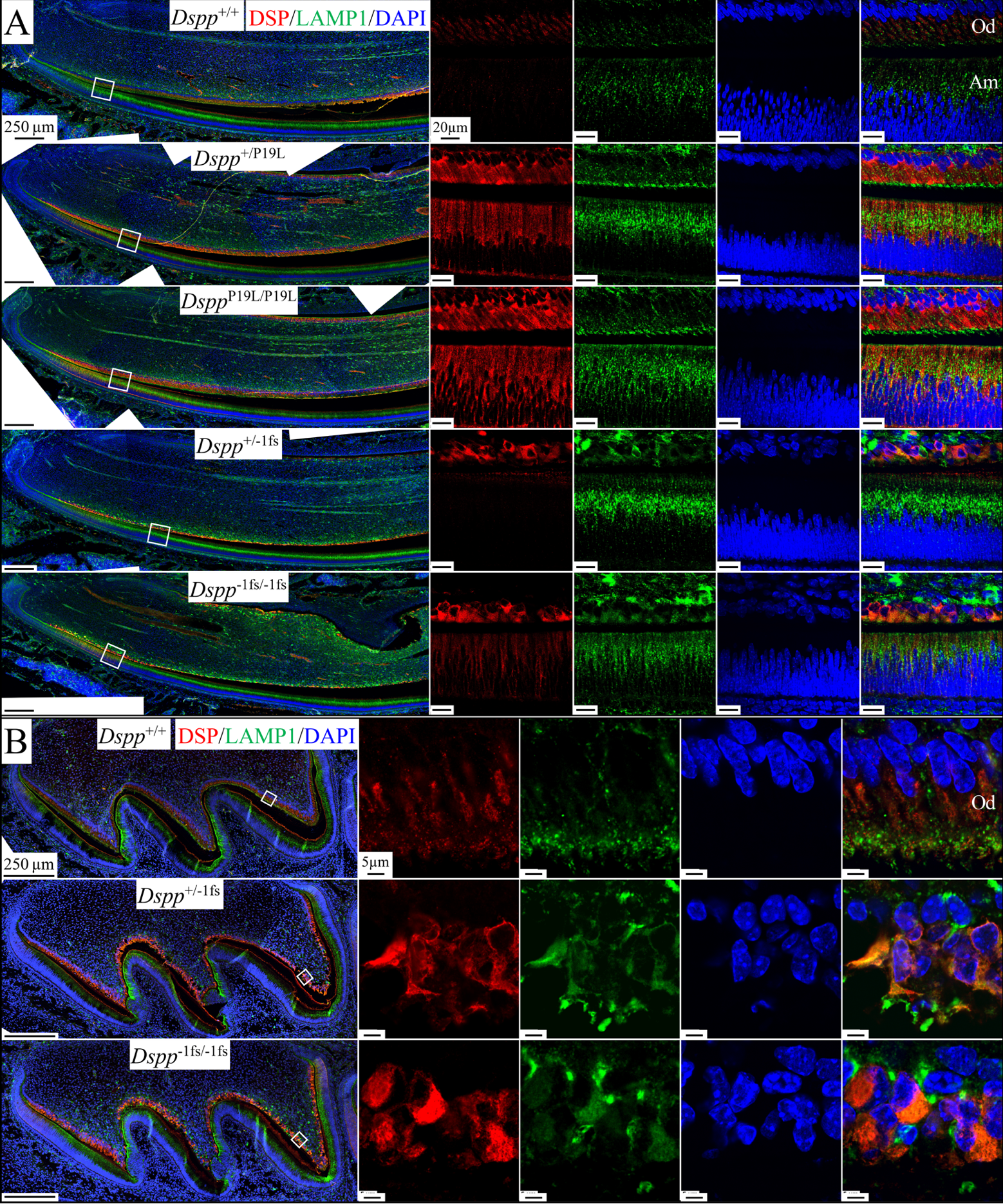


**Fig S5. Strong lysosome activities in** **the *Dspp*^-1fs^ odontoblasts.** Immunohistochemistry on D14 mandibular incisors (A) and D3 maxillary 1^st^ molars (B) of *Dspp*^P19L^ and *Dspp*^-1fs^ mice, showing DSP (red), LAMP1 (green; lysosomes), and DAPI (blue; nucleus). Lysosomes in *Dspp*^+/+^ and *Dspp*^P19L^ odontoblasts appear as small dots at distal region near the odontoblastic processes, not colocalizing with DSP signals. In odontoblasts and the cells in the sub-odontoblast layer of *Dspp*^-1fs^ mice, LAMP1 signals are higher in intensity, become less discrete and fuse into large plaques, indicating strong lysosome activities. LAMP1 and DSP signals partially overlap. In ameloblasts of all mice, LAMP1-positive organelles distribute at the supranuclear region, and weaker at the distal pole of the cell. Scale bar in A: 250(left)/20(right) μm. Scale bar in B: 250(left)/5(right) μm. Od, odontoblast; Am, ameloblast.

**
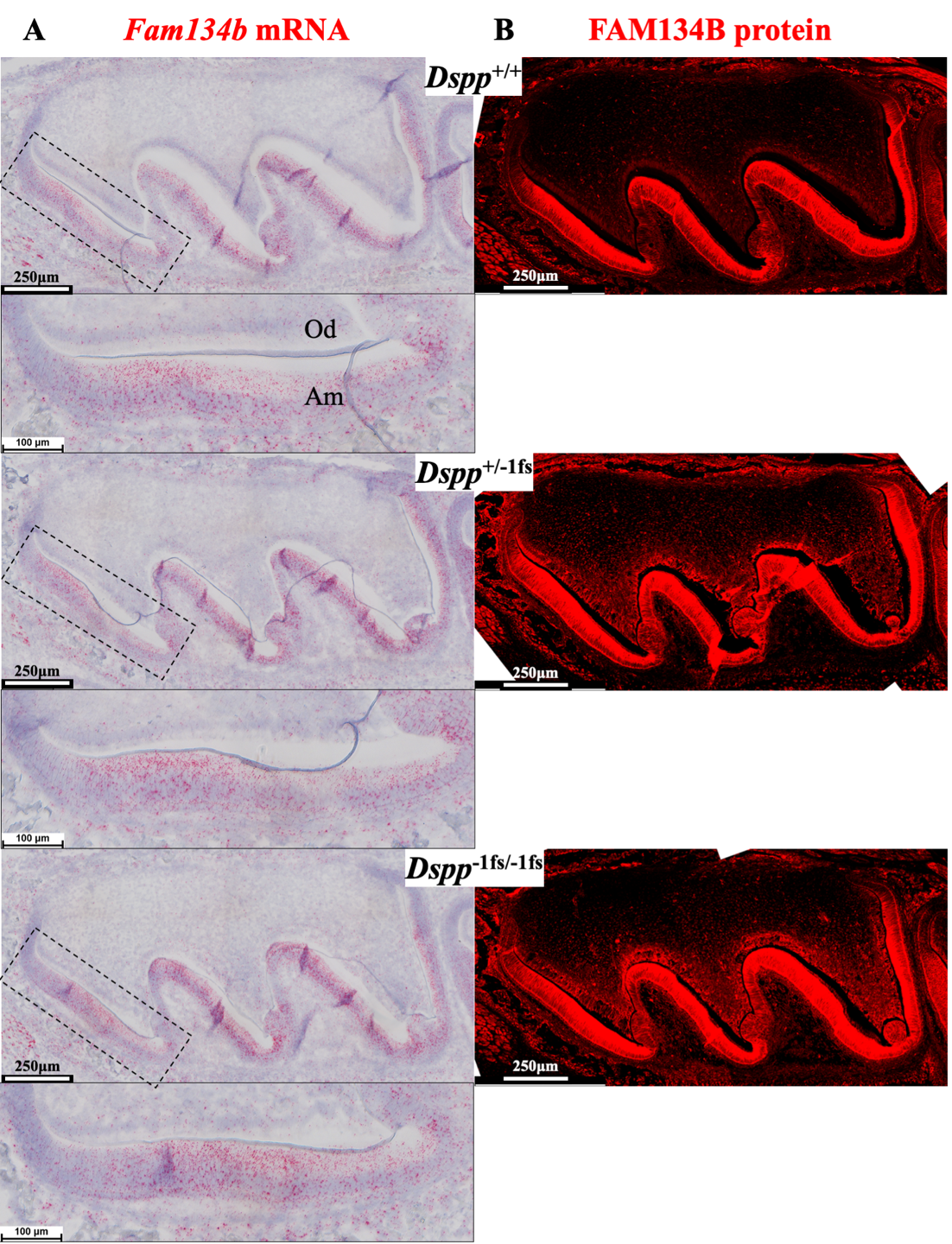
**

**Fig S6.** **Fam134b expression in *Dspp*^-1fs^ odontoblasts.** *In situ* hybridization showing *Fam134b* mRNA (A) and immunohistochemistry showing FAM134B protein (B) on D3 maxillary 1^st^ molars of *Dspp*^-1fs^ mice. **A.** *Fam134b* signal is broadly expressed as shown in red. Boxed areas are magnified. Secretory stage ameloblasts (Am) express *Fam134b* at a higher level than odontoblasts (Od). No obvious change of *Fam134b* expression is noted in the pathological odontoblasts in *Dspp*^-1fs^ mice. Scale bar: 250 (upper)/100 (lower) μm. **B.** Signal pattern of FAM134B protein matches that of *Fam134b* mRNA. FAM134B protein congregates in *Dspp*^-1fs^ odontoblasts. Refer to Fig 9A for higher magnification of signals. Scale bar: 250 μm.


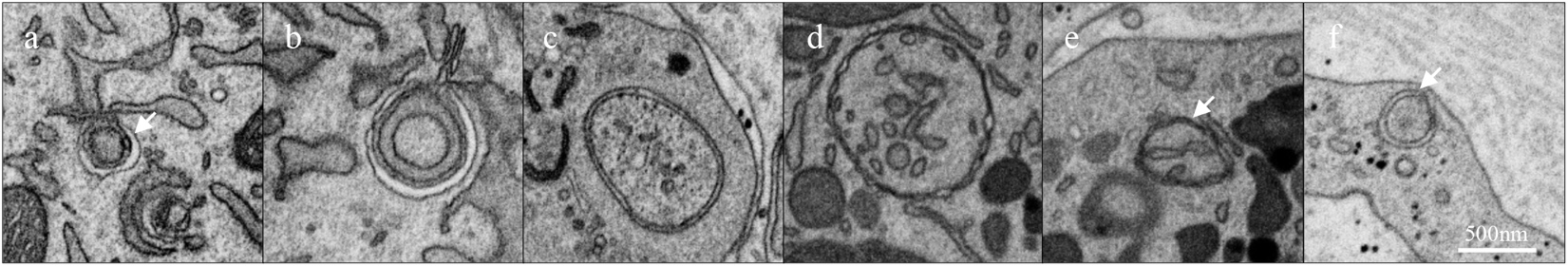


**Fig S7. Double-membrane-bounded autophagic structures in** ***Dspp*^-1fs^ odontoblasts.** Six double-membrane-bounded autophagic structures are identified from Areas -3 to S4 in *Dspp*^-1fs^ odontoblasts (annotation in Fig S2A). Inset a is from Area -1, b and c are from Area S1, d and e are from Area S2, and f is from Area S3. No double-membrane-bounded autophagic structure is noted from Areas -3 to S4 in WT odontoblasts (annotation in Fig S1A). Scale bar: 500 nm.
